# Valve thrombosis and antithrombotic therapy after bioprosthetic mitral valve replacement: a systematic review and meta-analysis

**DOI:** 10.1093/ehjcvp/pvaf005

**Published:** 2025-02-04

**Authors:** Mark J Zorman, Jonathan Vibhishanan, Katerina Dangas, James Castle, Ka Hou Christien Li, Marco Coronelli, Kate Eastwick-Jones, Alexander Swan, Nicky Johnson, Anurag Choksey, Helen Yan, Sam G C Scott, Matthew Henry, Mark Philip Cassar, Cara Barnes, Joao Ferreira-Martins, James Newton, Sam Dawkins, Mohamad Alkhouli, Charanjit Rihal, Mackram F Eleid, Sorin V Pislaru, Mayra E Guerrero, Jose Ordonez-Mena, Thomas J Cahill

**Affiliations:** Oxford Heart Centre, Oxford University Hospitals NHS Trust, Headley Way, Oxford, OX3 9DU, UK; Department of Cardiovascular Medicine, Mayo Clinic, 200 First Street SW, Rochester, Minnesota 55905, USA; Oxford Heart Centre, Oxford University Hospitals NHS Trust, Headley Way, Oxford, OX3 9DU, UK; Oxford Heart Centre, Oxford University Hospitals NHS Trust, Headley Way, Oxford, OX3 9DU, UK; Oxford Heart Centre, Oxford University Hospitals NHS Trust, Headley Way, Oxford, OX3 9DU, UK; Oxford Heart Centre, Oxford University Hospitals NHS Trust, Headley Way, Oxford, OX3 9DU, UK; Oxford Heart Centre, Oxford University Hospitals NHS Trust, Headley Way, Oxford, OX3 9DU, UK; Oxford Heart Centre, Oxford University Hospitals NHS Trust, Headley Way, Oxford, OX3 9DU, UK; Oxford Heart Centre, Oxford University Hospitals NHS Trust, Headley Way, Oxford, OX3 9DU, UK; Oxford Heart Centre, Oxford University Hospitals NHS Trust, Headley Way, Oxford, OX3 9DU, UK; Oxford Heart Centre, Oxford University Hospitals NHS Trust, Headley Way, Oxford, OX3 9DU, UK; Oxford Heart Centre, Oxford University Hospitals NHS Trust, Headley Way, Oxford, OX3 9DU, UK; Oxford Heart Centre, Oxford University Hospitals NHS Trust, Headley Way, Oxford, OX3 9DU, UK; Health Care Libraries, University of Oxford, John Radcliffe Hospital, Headley Way, Oxford, OX3 9DU, UK; Oxford Heart Centre, Oxford University Hospitals NHS Trust, Headley Way, Oxford, OX3 9DU, UK; Oxford Heart Centre, Oxford University Hospitals NHS Trust, Headley Way, Oxford, OX3 9DU, UK; Oxford Heart Centre, Oxford University Hospitals NHS Trust, Headley Way, Oxford, OX3 9DU, UK; Oxford Heart Centre, Oxford University Hospitals NHS Trust, Headley Way, Oxford, OX3 9DU, UK; Oxford Heart Centre, Oxford University Hospitals NHS Trust, Headley Way, Oxford, OX3 9DU, UK; Department of Cardiovascular Medicine, Mayo Clinic, 200 First Street SW, Rochester, Minnesota 55905, USA; Department of Cardiovascular Medicine, Mayo Clinic, 200 First Street SW, Rochester, Minnesota 55905, USA; Department of Cardiovascular Medicine, Mayo Clinic, 200 First Street SW, Rochester, Minnesota 55905, USA; Department of Cardiovascular Medicine, Mayo Clinic, 200 First Street SW, Rochester, Minnesota 55905, USA; Department of Cardiovascular Medicine, Mayo Clinic, 200 First Street SW, Rochester, Minnesota 55905, USA; Nuffield Department of Primary Care Sciences, University of Oxford, Radcliffe Observatory Quarter, Woodstock Road, OX2 6GG, UK; Oxford Heart Centre, Oxford University Hospitals NHS Trust, Headley Way, Oxford, OX3 9DU, UK

**Keywords:** Transcatheter mitral valve replacement, Surgical mitral valve replacement, Valve thrombosis

## Abstract

**Aims:**

Transcatheter mitral valve replacement (TMVR) has become a feasible alternative to surgical mitral valve replacement (SMVR) in selected patients at high surgical risk. The risk of valve thrombosis following SMVR and TMVR, and the optimal antithrombotic therapy following these procedures, remains uncertain. We aimed to compare the incidence of bioprosthetic mitral valve thrombosis (bMVT) after SMVR and TMVR, and the incidence of bMVT between patients on different antithrombotic regimens.

**Methods and results:**

A literature search of Medline, Embase, and Cochrane Library was performed between January 2000 and August 2024. Random-effects models were used to derive pooled estimates of the incidence of bMVT in the absence of prior or active endocarditis and valve thrombosis. A total of 47 studies (6170 patients, total follow-up 9541.8 patient-years) were eligible for inclusion. The overall incidence of bMVT was 5.05 [95% confidence interval (CI) 3.18–8.01, *I*^2^ = 82%] per 100-patient-years. Subclinical bMVT was more common than clinically significant bMVT: incidence 19.11 vs. 7.91 per 100-patient-years, adjusted incidence rate ratio (aIRR) 4.62 (95% CI 1.39–15.36), *P* = 0.012. bMVT was numerically more common after TMVR than SMVR, but the comparison was not statistically significant: incidence 7.03 vs. 0.58 per 100-patient-years, aIRR 2.19 (95% CI 0.72–6.72), *P* = 0.170. Patients on vitamin-K antagonists (VKA) had a lower incidence of bMVT than patients on direct oral anticoagulants (DOAC; incidence 5.72 vs. 17.08, aIRR 0.31, 95% CI 0.13–0.73, *P* = 0.007).

**Conclusions:**

bMVT is not uncommon, with numerically higher incidence in transcatheter compared to surgical valves, but the comparison was not statistically significant. VKAs are associated with a lower incidence of bMVT compared to DOACs.

## Graphical Abstract

**Graphical Abstract:**
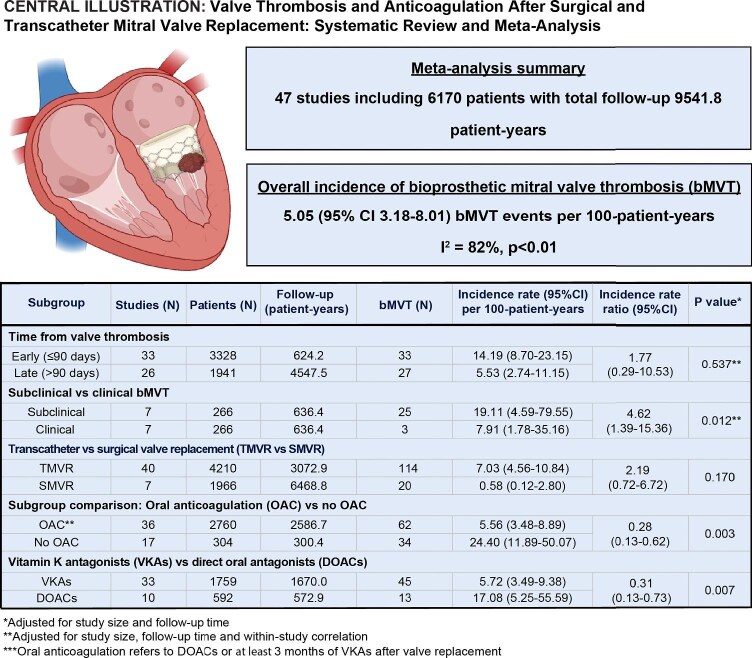
Valve thrombosis and antithrombotic therapy after surgical and transcatheter mitral valve replacement: a systematic review and meta-analysis. Created with Biorender.com.

## Introduction

Disease of the mitral valve is common, underdiagnosed, and associated with significant morbidity and mortality.^1,2^ While surgical repair and replacement remains the gold standard for treating native mitral valve disease, up to half of patients with significant mitral regurgitation are ineligible for surgery due to high burden of comorbidities.^1,3^ The challenge is even greater in patients with degenerated mitral bioprostheses, failed rings, or severe mitral annular calcification (MAC), where risks profiles commonly prohibit surgical (re)intervention.^4,5^ In selected patients with suitable anatomy and high or prohibitive surgical risk, transcatheter mitral valve replacement (TMVR) has become a feasible alternative to surgical mitral valve replacement (SMVR).

Bioprosthetic mitral valve thrombosis (bMVT) is significant complication of mitral valve replacement. Current guidelines recommend 3–6 months of oral anticoagulation (OAC) with vitamin K antagonists (VKA) after bioprosthetic SMVR, based on observational series suggesting a higher incidence of clinical bMVT after SMVR compared to aortic valve replacement.^6–11^ The need for anticoagulation has been recognized in patients with TMVR, but the incidence of bMVT after TMVR compared to SMVR, and between patients on different anticoagulation regimens, have not been defined.^12^ After transcatheter aortic valve replacement, there is a recognition that subclinical leaflet thrombosis is common, associated with embolism, and may be a precursor of structural valve degeneration.^13,14^

The aims of this study were (i) to determine the overall incidence of clinical and subclinical bMVT in patients after bioprosthetic mitral valve replacement, (ii) to evaluate the timing of bMVT after bioprosthetic mitral valve replacement, (iii) to compare the incidence of bMVT after SMVR and TMVR, and (iv) between patients taking vitamin-K antagonists (VKAs) versus direct oral anticoagulants (DOACs).

## Methods

### Search strategy

The protocol of this systematic review and meta-analysis has been registered in the International Prospective Register of Systematic Reviews (PROSPERO, CRD42024538972) and the reporting of this systematic review is informed by the meta-analysis of observational studies in epidemiology reporting guidelines (Supplementary Material 1).^15^ Embase (Ovid), MEDLINE (Ovid), Cochrane Central Register of Controlled Trials (CENTRAL), and Cochrane Database of Systematic Reviews were searched with a date range 2000–2024 (date of the original search 9 October 2023; last repeated on 25 August 2024 to identify new publications; Supplementary Material 2). No language restrictions were used. Covidence (Veritas Health Innovation, Melbourne, Australia) was used to deduplicate the search results. The reference lists of prior systematic reviews and included publications were screened to identify additional potentially relevant studies.

### Inclusion and exclusion criteria

Studies were included according to the following criteria: (i) adult patients (age ≥18 years) who underwent (ii) surgical bioprosthetic mitral valve replacement (SMVR), or (iii) TMVR. The TMVR group included all TMVR modalities: valve in native non-calcified valve, valve-in-bioprosthetic-valve, valve-in-ring, and valve-in-mitral-annular-calcification. Studies were excluded if they met any of the following criteria: (i) mechanical mitral valve replacement, (ii) surgical or transcatheter mitral valve repair, i.e. annuloplasty or edge-to-edge repair, (iii) active or prior infective endocarditis or valve thrombosis, (iv) pregnancy, (v) congenital or acquired coagulation disorders, (vi) studies with insufficient data to ascertain the incidence of bMVT (including cross-sectional studies, case reports, case series with <3 patients, surgical explant studies), and (vii) studies with no reported echocardiography or computed tomography (CT) surveillance to identify bMVT. Study eligibility was independently assessed by two reviewers, and disagreements were resolved by a third reviewer. In case of studies reporting on the same patient cohort, we included data from the most recent comprehensive cohort. This study required no ethical approval.

### Outcome

The primary outcome was the incidence of clinical or subclinical bMVT. Subclinical bMVT was defined as valve dysfunction with imaging evidence of valve thrombosis (i.e. overt thrombosis, hypoattenuated leaflet thickening or restricted leaflet motion on CT, and overt thrombosis or hypoechogenic valve leaflet thickening on echocardiography) in the absence of clinical symptoms.^16^ Clinical bMVT was defined as valve dysfunction with imaging evidence of valve thrombosis in a symptomatic patient.^16^

### Data extraction

Study screening and data extraction phases of the review were completed using an electronic database in Covidence systematic review software (Veritas Health Innovation, Melbourne, Australia). Data from included studies were independently extracted by two investigators. Inconsistencies were resolved by a third reviewer. Inter-investigator reliability was evaluated using Kohen's κ coefficient. The extracted data included patient baseline characteristics, sample size, the type of mitral valve intervention, number and timing of bMVT events, anticoagulation regimen, and follow-up time.

### Statistical analysis

The number of bMVT events and follow-up time were used to calculate study-specific incidence rates (IRs). To account for failure of valve implantation, mortality and loss to follow-up, individual patient data or the number of observations at follow-up were used for analysis where available. Meta-analysis methods were used to combine log-transformed IRs using a random effects model with the between-studies variance estimated by the maximum-likelihood method. A continuity correction of 0.5 was applied to studies with zero events. Heterogeneity was assessed using the Cochran Q statistic and *I*^2^ values.^17^ Meta-regression was used to identify significant covariates and adjust for their impact on the incidence of bMVT. Subgroup analyses were conducted to explore the impact of different valve interventions and antithrombotic regimens on the incidence of bMVT. Adjustment for within-study correlation based on block-diagonal variance-covariance matrix (correlation coefficient 0.5) was used in subgroup comparisons of non-independent bMVT events (early/late, subclinical/clinical events) recorded in the same patient population. bMVT events in patients with subtherapeutic anticoagulation or medication non-compliance were excluded from subgroup comparisons of anticoagulation regimens. The results were reported as incidence rate ratios (IRRs) with 95% confidence intervals (CIs). Measurable confounding in subgroup comparisons was evaluated using meta-analysis of patient background characteristics (random effects model, maximum-likelihood estimation of between-study variance). Publication bias and small-study effect were assessed by visual inspection of funnel plots and by Egger's test. Baujat plots were used to explore study contribution to overall heterogeneity and their impact on the incidence of bMVT.^18^ Leave-one-out sensitivity analyses were conducted to assess how each study affects the overall effect size estimates. Statistical significance was set at a *P* value <0.05 (2-sided). All analyses were performed with R, version 4.4.0 (R Foundation for Statistical Computing), packages meta and metafor.^19–21^

## Results

### Study inclusion

**Figure 1:**
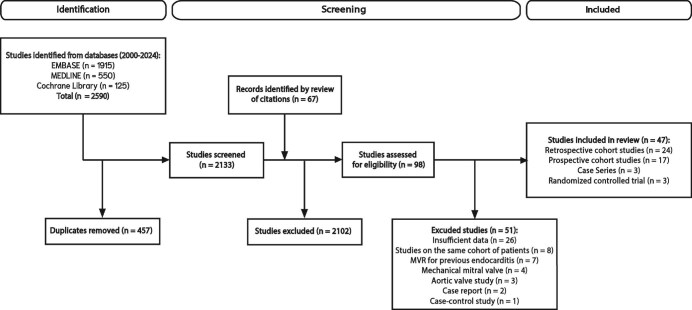
Study flow diagram.

From a total of 2590 studies identified by the literature search, 47 studies including a total of 6170 patients met the eligibility criteria for inclusion (*Figure 1*, *Table 1*, Kohen's κ 0.72). Patient baseline characteristics from included studies are summarized in Supplementary Material 3: the median age was 73.5 years and 46.1% of patients were male.

**Table 1:** Study characteristics

First author, Year	Study design	*N* (centres)	Study period	Intervention (valve)	*N* (patients)	Follow-up imaging modality
Akodad (2023)	Retrospective	1	2008–2021	TMVR (53 Sapien XT, 52 Sapien 3, 12 Cribier, 2 J-valve)	119	TTE
Alpieri (2020)	Case series	1	July 2019–October 2019	TMVR (3 Cephea)	3	TTE, CCTA
Altisent (2015)	Case series	1		TMVR (3 Fortis)	3	TTE, TEE, CCTA
Bourguignon (2014)*	Retrospective	1	August 1984–March 2011	SMVR (430 Perimount)	430	TTE
Bapat (2018)	Prospective			TMVR (50 Intrepid)	50	TTE
Brener (2024)	Retrospective	12	November 2014–May 2021	TMVR (3 Sapien, 118 Sapien 3, 6 Sapien XT)	126	TTE
Brennan (2012)	Retrospective	40	May 2007–August 2008	SMVR (44 Biocor, 9 Epic)	53	TTE
Butnaru (2013)	Retrospective	1	January 2002–December 2011	SMVR (139 Hancock II, 4 Epic, 6 Perimount)	149	TTE
Capretti (2016)	Retrospective	1		TMVR (70 Sapien 3 or XT)	70	TTE, TEE, CCTA
Cheung (2013)	Prospective	1	July 2007–September 2012	TMVR (23 Sapien or Sapien XT)	23	TTE
Conradi (2024)	Prospective	36	November 2014–June 2020	TMVR (191 Yendyne)	191	TTE
Da Costa (2020)	Prospective	1	May 2015–July 2018	TMVR (50 Braile Inovare)	50	TTE
Dahle (2017)	Retrospective	1	November 2011–September 2016	TMVR (1 Sapien 3, 7 Sapien XT, 1 Lotus, 2 Tendyne)	11	TTE
Duncan (2017)	Case series	1	October 2014–September 2015	TMVR (5 Tendyne)	5	TTE
El Beze (2024)	Prospective	1	March 2011–March 2023	TMVR (156 Sapien)	156	TTE
Eleid (2017)	Retrospective	6	January 2014–March 2017	TMVR (87 Sapien, Sapien XT or Sapien 3)	87	TTE
Eng (2017)	Prospective	1	December 2013–December 2015	TMVR (12 Sapien, Sapien XT or Sapien 3)	13	TTE
Guerrero (2018)	Retrospective	51	September 2012–March 2017	TMVR (5 Sapien, 52 Sapien XT, 57 Sapien 3, 2 Inovare)	116	TTE
Guerrero (2020)**	Retrospective	172	March 2013–June 2017	TMVR (32 Sapien, 313 Sapien XT, 406 Sapien 3, 28 other/unknown)	780	TTE
Guerrero (2023)	Prospective	13	February 2015–December 2017	TMVR (91 Sapien 3)	91	TTE
Guerrero (2024)	Retrospective	236	August 2015–December 2022	TMVR (9 Sapien 3 Ultra Resilia, 227 Sapien 3 Ultra, 584 Sapien 3)	820	TTE
Guimarães (2020)	RCT	49	April 2016–July 2019	SMVR	1005	TTE
Gaia (2017)	Retrospective	1	June 2010–January 2013	TMVR (12 Braile Inovare)	12	TTE
Gwak (2022)	Retrospective	1	June 1996–May 2015	SMVR (149 Perimount, 60 Epic, 19 Biocor, 11 other))	239	TTE
Hosoba (2020)	Retrospective	1	2007–2017	SMVR (16 Magna Ease, 15 Mosaic, 12 Carpentier-Edwards, 10 St. Jude Epic)	53	TTE, CT
Kalil (2021)	Retrospective	1	May 2013–February 2020	TMVR (unknown)	31	TTE
Kawano (2021)	Retrospective	1	June 2018–November 2020	TMVR (10 Sapien 3)	11	TTE
Kuohn (2022)	Retrospective	1	January 2015–June 2021	TMVR (unknown)	57	TTE
Long (2018)	Retrospective	1	January 2013–December 2018	TMVR (24 Sapien XT or Sapien 3)	24	TTE
Ludwig (2021)	Retrospective	1	January 2016–December 2020	TMVR (7 Tendyne, 4 Tiara)	11	TTE
Ludwig (2023)	Retrospective	31	May 2014–July 2022	TMVR (1 AltaValve, 4 CardiAQ, 1 FORTIS, 7 HighLife, 23 Intrepid, 310 Tendyne, 28 Tiara, 2 Cardiovalve, 5 Cephea, 5 Evoque, and 14 Sapien M3)	400	TTE
Malaisrie (2024)	Prospective	12	2018–2021	TMVR (50 Sapien 3)	50	TTE
Mandiye (2022)	Retrospective	1	January 2018–December 2021	SMVR (37 Hancock II)	37	TTE
Praz (2018)	Prospective	6	April 2015–September 2017	TMVR (2 Sapien XT, 24 Sapien 3)	26	TTE
Regueiro (2017)	Prospective	5	February 2014–March 2015	TMVR (13 Fortis)	13	TTE
Rogers (2023) (1)	RCT	72	June 2018–ongoing	TMVR (100 Tendyne)	100	TTE
Rogers (2023) (2)	RCT	72	June 2018–ongoing	TMVR (97 Tendyne)	97	TTE
Schneider (2023)	Prospective	13	June 2019–July 2021	TMVR (30 HighLife)	30	TTE
Sorajja (2019)	Prospective	5		TMVR (9 Tendyne)	9	TTE
Ussia (2017)	Retrospective	1	March 2015–November 2015	TMVR (4 CardiAQ)	4	TTE, TEE
Webb (2019)	Prospective	1	August 2017–August 2018	TMVR (10 Sapien M3)	10	TTE, CCTA
Webb (2020)	Prospective	1	September 2018–October 2019	TMVR (14 Evoque)	14	TTE, CCTA
Wilbring (2014)	Prospective	1	November 2008–September 2014	TMVR (14 Sapien XT)	14	TTE
Wild (2022)	Retrospective	26	January 2020–April 2021	TMVR (108 Tendyne)	108	TTE
Ye (2015)	Prospective	1	April 2007–December 2013	TMVR (31 Sapien or Sapien XT)	31	TTE
Yoon (2019)	Retrospective	40	November 2015–April 2018	TMVR (27 Sapien, 154 Sapien XT, 288 Sapien 3, 30 Lotus, 18 Direct Flow, 4 Melody)	411	TTE
Zahr (2023)	Prospective	9	February 2020–August 2022	TMVR (33 Intrepid)	33	TTE, TEE, CCTA

TTE, transthoracic echocardiography; TEE, transesophageal echocardiography; and CCTA, coronary computed tomography angiogram.

* Patients with previous endocarditis were excluded from the analysis.

** Includes valve-in-valve and valve-in mitral-annular-calcification cohorts, valve thrombosis outcomes for valve-in-ring patients with longer follow-up are included in Guerrero et al. (2024)^38^.

### Incidence of bMVT

**Figure 2:**
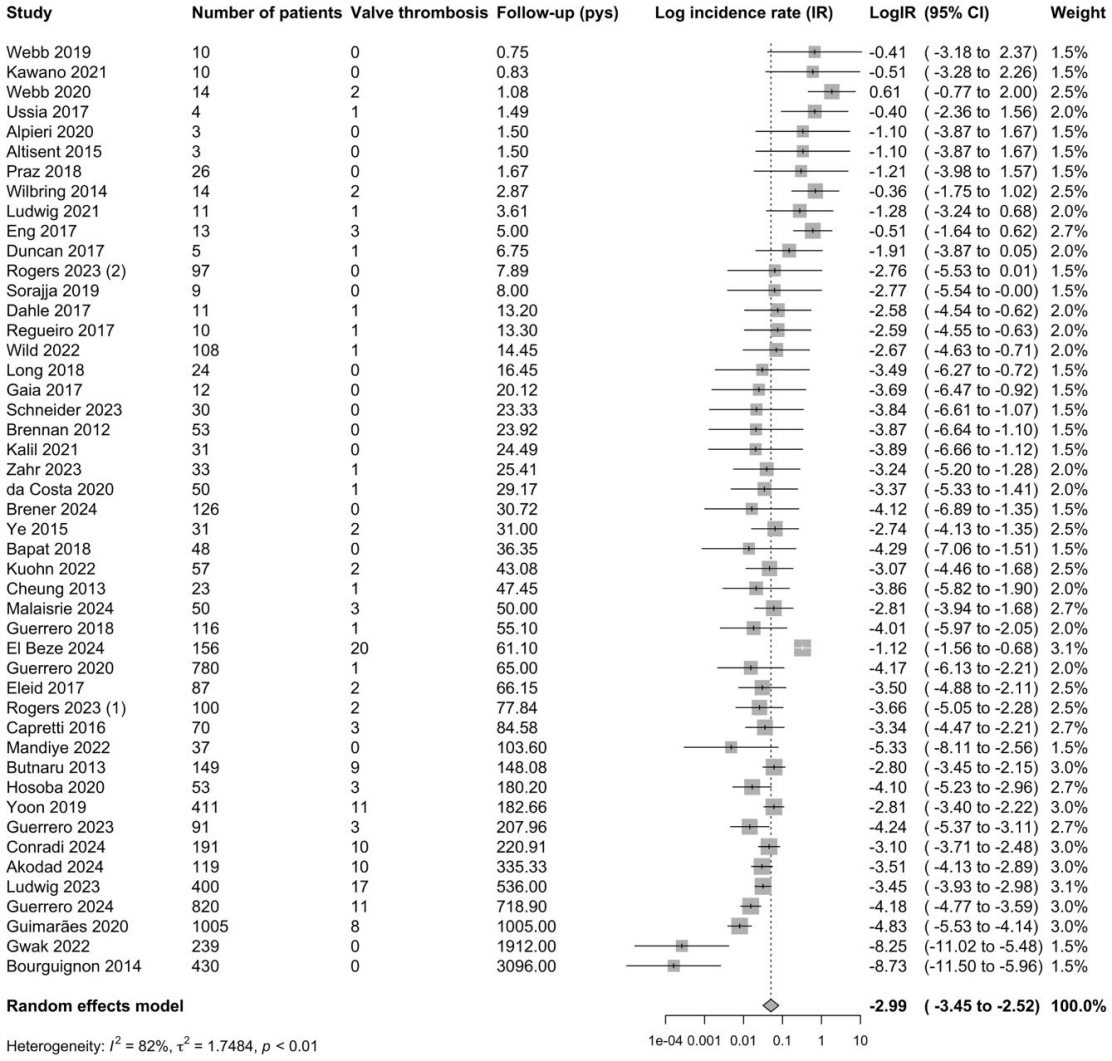
Forest plot of log incidence rate of bioprosthetic mitral valve thrombosis. Studies are presented in ascending order of follow-up (pys, patient-years). IR, incidence rate and 95% CI, 95% confidence intervals.

A total of 6170 patients had a follow-up duration of 9541.8 patient-years, with a median study follow-up time of 15.5 months (95% CI 9.1–21.9 months). The total number of bMVT events was 134. The pooled incidence of bMVT across all studies was 5.05 (95% CI 3.18–8.01) per 100-patient-years, *I*^2^ = 82%, *P* < 0.01; *Figure 2*, *Table 2*). Univariate meta-regression analysis revealed a significant impact of follow-up time, sex, diabetes, and chronic kidney disease (CKD) on the incidence of bMVT; the impact of follow-up time remained significant after multivariate adjustment (Supplementary Material 4).

**Table 2:** Summary of meta-analysis results

	Studies (*N*)	Patients (*N*)	Follow-up (patient-years)	bMVT events (*N*)	Incidence rate (95% CI) per 100-patient-years	Incidence rate ratio (95% CI)*	Heterogeneity	*P* value*
Overall								
All studies	47	6170	9541.8	134	5.05 (3.18–8.01)		*I* ^2^ = 82%, τ^2^ = 1.75	
By time from valve replacement								
Early (≤90 days)	33	3328	624.2	33	14.19 (8.70–23.15)	Early/late: 1.77 (0.29–10.53)	*I* ^2^ = 66%, τ^2^ = 1.61	0.537 **
Late (>90 days)	27	1941	4547.5	34	5.53 (2.74–11.15)			
bMVT type: subclinical vs. clinical								
Subclinical	7	266	317.4	25	19.11 (4.59–79.55)	Subclinical/clinical: 4.55 (1.39–15.36)	*I* ^2^ = 84%, τ^2^ = 3.60	0.012**
Clinical	7	266	317.4	3	7.91 (1.78–35.16)			
Valve intervention: TMVR vs. SMVR								
TMVR	40	4210	3072.9	114	7.03 (4.56–10.84)	TMVR/SMVR: 2.19 (0.72–6.72)	*I* ^2^ = 82%, τ^2^ = 1.75	0.170
SMVR	7	1966	6468.8	20	0.58 (0.12–2.80)			
Oral anticoagulation								
OAC***	36	2760	2586.7	62	5.56 (3.48–8.89)	OAC/No OAC: 0.28 (0.13–0.62)	*I* ^2^ = 79%, τ^2^ = 1.69	0.003
No OAC***	17	304	300.4	34	24.40 (11.89–50.07)			
VKAs vs. DOACs								
VKAs	33	1759	1670.0	45	5.72 (3.49–9.38)	VKAs/DOACs: 0.31 (0.13–0.73)	*I* ^2^ = 71%, τ^2^ = 1.38	0.007
DOACs	10	592	572.9	13	17.08 (5.25–55.59)			

bMVT, bioprosthetic mitral valve thrombosis; TMVR, transcatheter mitral valve replacement; SMVR, surgical mitral valve replacement; OACs, oral anticoagulants; VKAs, vitamin K antagonists; DOACs, direct-acting oral anticoagulants; and 95% CI, 95% confidence intervals.

* Adjusted for follow-up time.

** Adjusted follow-up time and within-study correlation.

*** Oral anticoagulation refers to DOACs or ≥3 months of VKAs after valve replacement.

### Timing of bMVT after valve replacement

Valve thrombosis events were classified into early (occurring ≤90 days from valve replacement) and late (occurring >90 days from valve replacement). A total of 33 early bMVT events were reported in 33 studies on 3328 patients. A total of 34 late bMVT events were reported in 27 studies on 1941 patients (Supplementary Material 5). Unadjusted comparison showed a higher incidence of early compared to late bMVT events: incidence 14.19 (95% CI 8.70–23.15) compared to 5.53 (95% CI 2.74–11.15) per 100-patient-years, IRR 2.49 (95% CI 1.09–5.70), *P* = 0.030. After adjustment for follow-up time and within-study correlation, the rates were statistically comparable: adjusted incidence rate ratio (aIRR) was 1.77 (95% CI 0.29–10.53), *P* = 0.537 (Supplementary Material 5, *Table 2*).

### Subclinical vs. clinical bMVT

**Figure 3:**
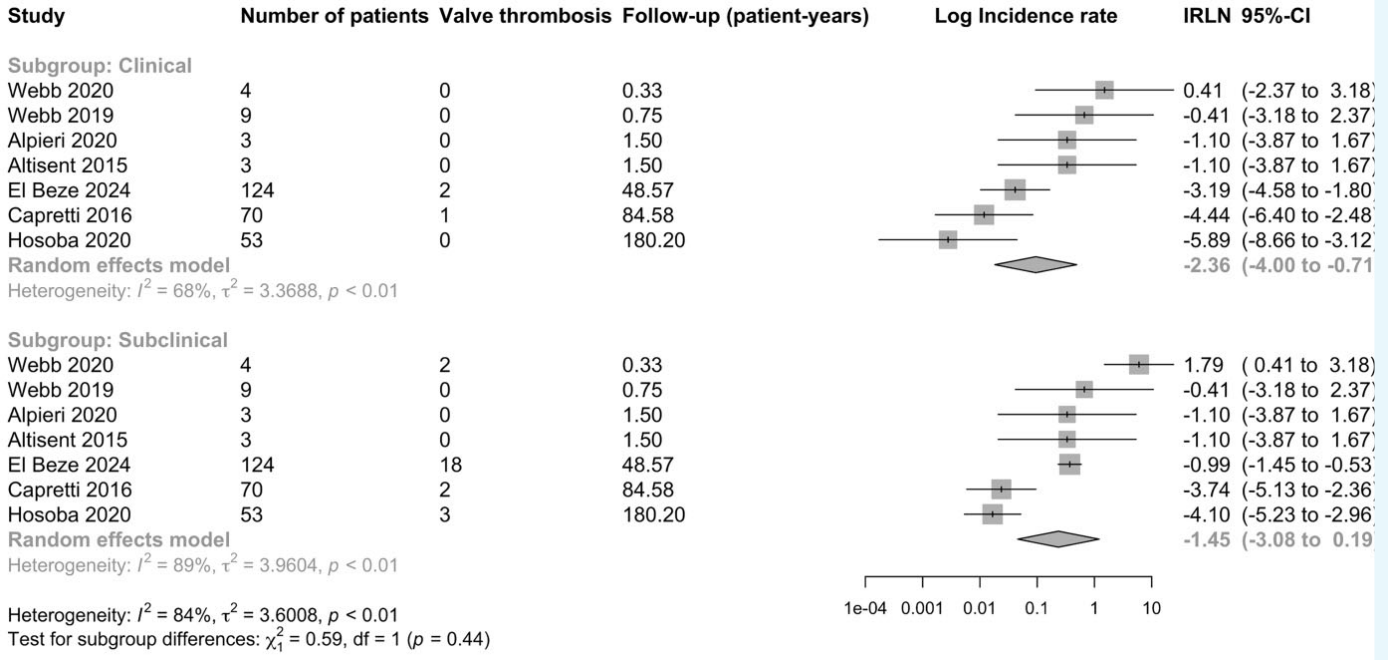
Forest plot of log incidence rate of subclinical versus clinical bMVT. The analysis only includes studies with reported CT follow-up after discharge. IR, incidence rate and 95% CI, 95% confidence intervals.

In addition to echocardiography, 15% (7/47) of studies also included cardiac CT follow-up (*Table 1*). These studies reported a total of 25 subclinical and 3 clinical bMVT events in 266 patients over 317.4 patient-years of follow-up (*Figure 3*). Unadjusted IRs of subclinical and clinical bMVT were comparable: incidence 19.11 (95% CI 4.59–79.55) vs. 7.91 (95% CI 1.78–35.16) per 100-patient-years, IRR 2.39 (95% CI 0.30–19.09), *P* = 0.410. Adjusted for follow-up time and within-study correlation, subclinical bMVT events were significantly more common than clinical bMVT events: aIRR 4.55 (95% CI 1.39–15.36), *P* = 0.012 (*Table 2*).

### Incidence of bMVT after TMVR vs. SMVR

**Figure 4:**
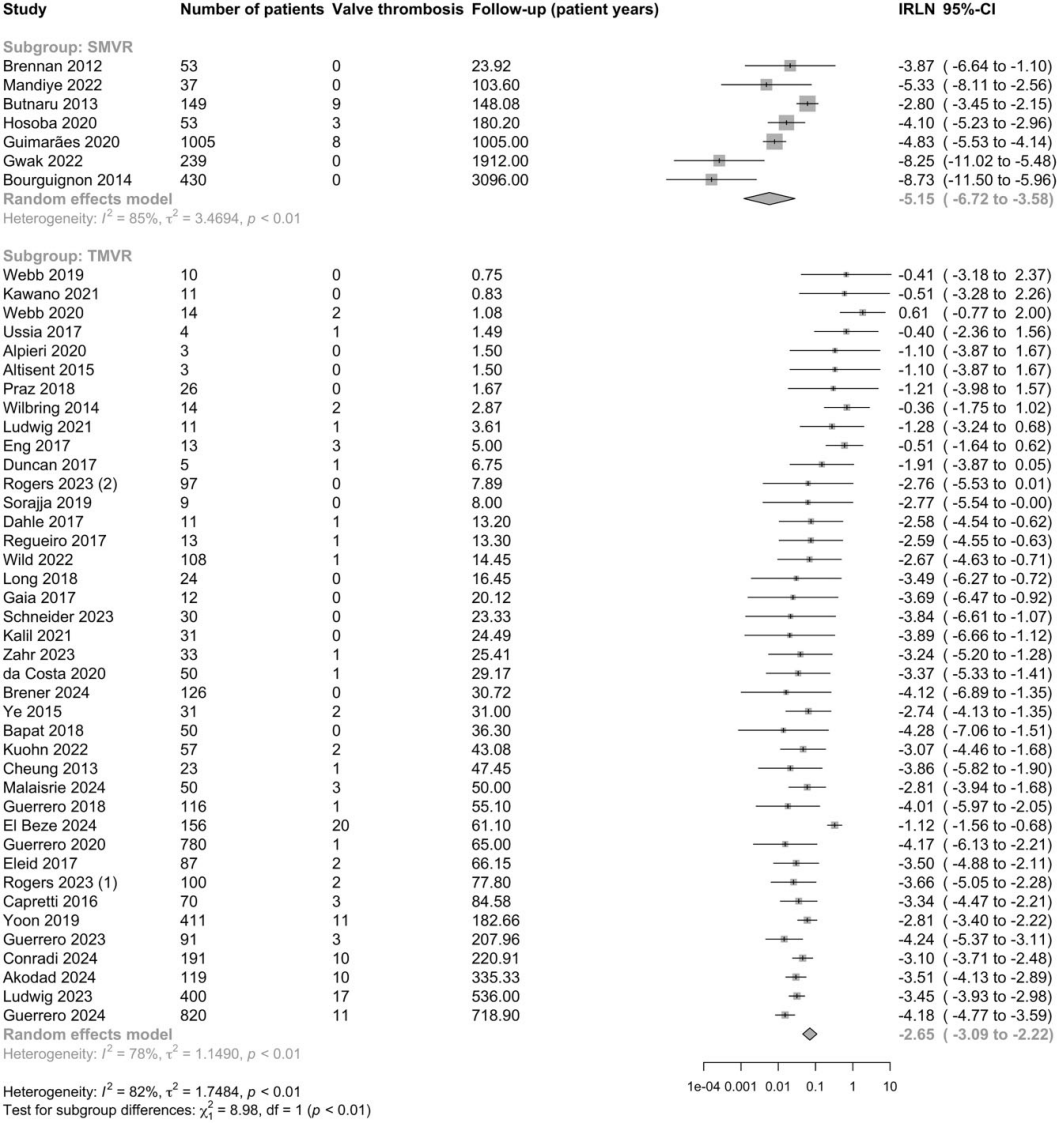
Forest plot of log incidence rate of bMVT after SMVR vs. TMVR. Studies are presented in ascending order of follow-up (patient-years). IR, incidence rate and 95% CI, 95% confidence intervals. The unadjusted comparison of SMVR and TMVR is statistically significant (*P* < 0.01), but the groups are comparable when adjusted for study size and follow-up time (*Table 2*).

A total of 114 TMVR valve thrombosis events were reported in 40 studies including 4210 patients. In comparison, a total of 20 SMVR valve thrombosis events were reported by 7 studies including 1966 patients (*Figure 4*). Unadjusted comparison showed a higher incidence of bMVT after TMVR compared to SMVR: IR 7.03 (95% CI 4.56–10.84) per 100-patient years compared to 0.58 (95% CI 0.12–2.80) per 100-patient-years, IRR 9.51 (95%CI 2.87–31.48), *P* < 0.001. After adjustment for follow-up time, the IRs of bMVT after TMVR and SMVR were statistically equivalent: aIRR 2.19 (95% CI 0.72–6.72), *P* = 0.170 (*Table 2*). The pooled SMVR cohort was younger, with lower diabetes and CKD rates than the TMVR cohort (Supplementary Material 3).

### OAC after mitral valve replacement

**Figure 5:**
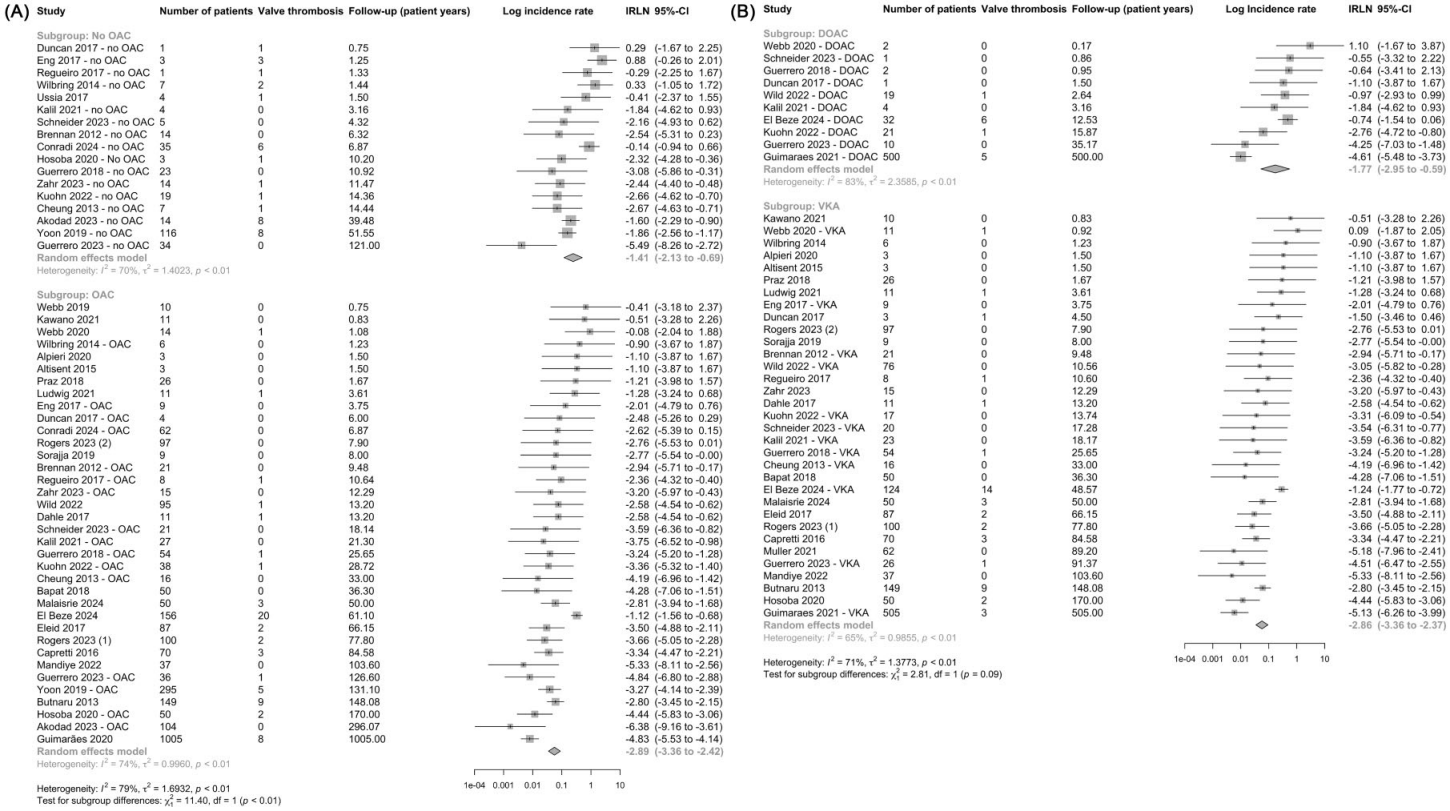
Oral anticoagulation after bioprosthetic mitral valve replacement. (*A*) Forest plot of log incidence rate of bMVT in patients taking anticoagulation vs. patient without. (*B*) Forest plot of log incidence rate of bMVT in patients taking DOACs vs. VKAs. Studies are presented in ascending order of follow-up (patient-years). IR, incidence rate and 95% CI, 95% confidence intervals.

Thirty six studies including a total of 2760 patients on OAC (defined as use of a DOAC or at least 3 months of VKA after valve replacement) reported a total of 62 bMVT events. In comparison, 17 studies on a total of 304 patients with no OAC reported a total of 34 bMVT events (*Figure 5A*). No OAC cohort included patients on no antithrombotic medications, single and dual antiplatelet therapy. Patients on OAC had a 3.5-fold lower incidence of bMVT compared to patients without: IR 5.56 (95% CI 3.48–8.89) compared to 24.40 (95% CI 11.89–50.07) per 100-patient-years, aIRR 0.28 (95% CI 0.13–0.62), *P* = 0.002 (*Figure 5A*, *Table 2*).

Next, we analysed the incidence of bMVT in patients on VKAs compared to DOACs. A total of 10 studies included 592 patients on DOACs, which reported a total of 13 bMVT events (*Figure 5B*). In comparison, 33 studies including 1759 patients on VKAs (duration ≥ 3 months after valve replacement) reported a total of 45 bMVT events (*Figure 5B*). Details on the duration of VKA therapy and target international normalized ratios are provided (Supplementary Material 6). Patients on VKAs had a 3.2-fold lower incidence of bMVT compared to patients on DOACs: incidence 5.72 (95% CI 3.49–9.38) compared to 17.08 (95% CI 5.25–55.59) per 100-patient-years, aIRR 0.31 (95% CI 0.13–0.73), *P* = 0.007 (*Figure 5B*, *Table 2*). The pooled DOAC cohort was younger, with lower diabetes and coronary artery disease and CKD rates than the VKA cohort (Supplementary Material 3).

### Publication bias and sensitivity analyses

Visual inspection of funnel plots and results from Egger's test indicated no significant publication bias (*P* > 0.05). After reviewing studies with significant heterogeneity and/or effect size impact based on Baujat plots, no studies were excluded. Funnel plots, Baujat plots and influential analyses for each subgroup comparison are provided (Supplementary Material 7). In analyses requiring adjustment for within-study correlation (early/late, subclinical/clinical bMVT events), varying the correlation coefficient (range 0.20–0.80) did not impact subgroup comparisons.

## Discussion

This study is a systematic and comprehensive analysis of anticoagulation practices and the incidence of valve thrombosis after bioprosthetic mitral valve replacement. Our main findings are as follows: (i) bMVT occurred with an overall incidence of 5.05 events per 100-patient-years; (ii) bMVT events were numerically more common in the early (≤90 days) compared to late (>90 days) period after valve replacement but the rates were statistically comparable; (iii) the incidence of bMVT was higher in transcatheter compared to surgical bioprostheses but the comparison did not reach statistical significance; (iv) patients taking oral anticoagulation had a significantly lower incidence of bMVT compared to patients without; and (iv) VKA were associated with a significantly lower rate of bVMT than DOACs.

### Bioprosthetic mitral valve thrombosis

To our knowledge, this is the first systematic review and meta-analysis to evaluate the incidence of bMVT. Individual study estimates ranged from 0.02 to 92.32 per 100-patient-years, highlighting significant between-study heterogeneity likely affected by variable frequency and modality of screening. The overall incidence of bMVT in this analysis was 5.05 events per 100 patient-years, considerably higher than equivalent estimates for aortic bioprostheses reported at 1.2–3.0 valve thrombosis events per 100-patient years.^22,23^ Collectively, these data suggest a higher incidence of bioprosthetic valve thrombosis in the mitral compared to the aortic position, likely due to a greater surface area and lower pressure gradients. The FDA-mandated CT angiographic substudies of the PARTNER 3 and Low-Risk CoreValve Evolut trials revealed significantly higher rates of subclinical leaflet thrombosis compared to prior literature.^24,25^ As most studies in our analysis included only TTE surveillance, our pooled bMVT rate likely remains underestimated.

### Timing of bioprosthetic valve thrombosis

Current guidelines on antithrombotic therapy after bioprosthetic mitral valve replacement provide a class IIa recommendation for 3–6 months of oral anticoagulation with VKAs (INR target 2.5) during the period of endothelialization of the bioprosthesis.^6,7,26^ While an early study highlighted high frequency of bioprosthetic valve thrombosis in first 3 months after implantation,^10^ a number of subsequent studies demonstrated that the risk of valve thrombosis persists beyond 3 months and peaks at 12–24 months after valve implantation, a finding replicated in both mitral and aortic valves.^9,27^ Our analysis found a numerically higher incidence of bMVT in the early (≤90 days) compared to the late (>90 days) period after valve replacement, but the comparison was not statistically significant. While likely affected by higher frequency of screening in the early compared to the late period, these results reflect a significant risk of bMVT that persists beyond the period of valve endothelialization.

### Subclinical vs. clinical bMVT

In aortic bioprostheses, four-dimensional CT has been established as the most sensitive imaging modality for detecting hypoattenuated leaflet thickening and immobility.^24,25^ These findings typically resolve with anticoagulation, supporting the hypothesis that they represent subclinical valve thrombosis.^28^ Little is known about subclinical valve thrombosis in mitral bioprostheses. Based on 15% (7/47) of studies that reported CT imaging follow-up, our analysis found the pooled incidence of subclinical bMVT to be 4.6-fold higher than the incidence of clinical bMVT. Abnormal transvalvular gradients, detectable as early as 4 months before clinical diagnosis of bMVT, indicate a window for earlier diagnosis and treatment.^29^ However, the interpretation of raised transvalvular gradients may be challenging, and wider use of cardiac CT may improve early detection of subclinical bMVT. Reduced leaflet motion observed as early as at the time of valve implantation has been associated with early bioprosthetic degeneration, suggesting a role for subclinical thrombosis in linking cusp immobility and prosthetic failure.^30^

### Surgical vs. transcatheter valves

The PARTNER 3 and CoreValve trials reported similar valve thrombosis rates between surgical and transcatheter aortic bioprostheses,^24,25^ but comparisons of surgical and transcatheter mitral bioprostheses remain scarce. The distinct physical properties of surgical and transcatheter valves may impact their thrombosis rates. Transcatheter valve leaflets are typically thinner and more rigidly mounted within the stent.^31^ Both the neosinus and the sewing ring may promote clot formation, and suboptimal expansion of transcatheter bioprostheses against calcified native aortic valve tissue has been associated with higher rates of hypoattenuated leaflet thickening.^9,32^ In mitral annular calcification, asymmetric or incomplete stent frame expansion of balloon-expandable valves may cause leaflet motion abnormalities and predispose to bMVT.^33^ Our analysis found a numerically higher rate of bMVT after TMVR compared to SMVR. The SMVR cohort was younger and had significantly less CKD, though other baseline characteristics were comparable. After adjusting for follow-up time, bMVT rates were statistically similar but heterogeneous, highlighting the need for evaluation in a controlled, ideally randomized setting.

### Oral anticoagulation

Based on SMVR studies, current guidelines recommend 3–6 months of oral anticoagulation with VKAs after bioprosthetic mitral valve replacement.^6,7,9^ The need for anticoagulation has also been recognized in TMVR,^34,35^ but its impact beyond individual observational studies remains unclear.^12^ We found that patients on oral anticoagulation (DOACs or ≥3 months of VKAs) had a 3.6-fold lower incidence of bMVT compared to patients without. A large proportion of patients with severe mitral valve disease are already anticoagulated due to atrial fibrillation, but our study suggests that anticoagulation may be beneficial in all patients with bioprosthetic mitral valves for valve thrombosis. The net benefit of this approach requires consideration of the concomitant bleeding risk, which was beyond the scope of this study.

### VKAs vs. DOACs

Two previous studies have directly compared the impact of DOACs and VKAs on the incidence of bMVT. In SMVR patients, the RIVER trial demonstrated non-inferiority of rivaroxaban compared to warfarin with respect to all-cause mortality, valve thrombosis, and major bleeding.^36^ There were numerically more valve thrombosis events in the rivaroxaban arm compared to the warfarin arm (incidence 1.04 vs. 0.62 per 100-patient years, HR 1.68, 95% CI 0.40–7.01), but this did not reach statistical significance. A recent prospective study of 156 TMVR patients reported a comparable incidence of bMVT in patients on DOACs compared to VKA, although most thrombosis events in this series were subclinical.^37^ Our meta-analysis (2351 patients with follow-up of 2242.4 patient-years) showed a 3.2-fold lower incidence of bMVT with VKAs compared to DOACs. Patients taking DOACs were younger, with lower rates of diabetes and coronary artery disease than the VKA cohort, reducing the likelihood that our finding is confounded by variable patient demographics and comorbidities. In addition to the concern regarding renal clearance of DOACs in patients with advanced CKD, our study provides further evidence in favour of VKAs in patients undergoing TMVR. As randomized comparisons and bleeding data are lacking, optimal anticoagulation choice should remain individualized, incorporating each patient's relative risks of bleeding and bMVT.

### Limitations

The analysis is based on heterogeneous observational data with variability in patient background characteristics, imaging frequency and modalities. Meta-regression and meta-analysis tools were used to account for confounding with respect to valve thrombosis and patient background characteristics, but unmeasured confounding and selection bias in observational data cannot be excluded. Patients with prior infective endocarditis and valve thrombosis were excluded from the analysis, but incomplete reporting is a possibility. Overall, the findings are descriptive and hypothesis-generating, requiring validation in prospective and ideally randomized studies.

## Conclusions

bMVT is not uncommon, with statistically comparable IRs in both early (≤90 days) and late (>90 days) periods after valve replacement, and between surgical and transcatheter valves. Patients taking oral anticoagulation have a significantly lower incidence of bMVT than patients without. VKAs is associated with a significantly lower incidence of bMVT compared to DOACs. Further research is needed, and anticoagulation choice should remain individualized, balancing the risks of bleeding and valve thrombosis.

## Supplementary Material

pvaf005_Supplemental_Files

## Data availability

The data underlying this article are available in the article and in its online supplementary material.
