# Supplementary material for: Valve thrombosis and antithrombotic therapy after bioprosthetic mitral valve replacement: a systematic review and meta-analysis
Source: Eur Heart J Cardiovasc Pharmacother. 2025 Feb 4;11(3):251–63. doi: 10.1093/ehjcvp/pvaf005 (PMC12046575; doi:10.1093/ehjcvp/pvaf005)
Supplement: pvaf005_Supplemental_Files [file pvaf005_supplemental_files.zip › Supplement 7_Publication bias and sensitivity analyses.docx]

**Supplementary Material 7: Publication bias and sensitivity analyses**

1. **Overall incidence of bMVT**

**A)** Funnel plot exploring publication bias (Egger’s test, p=0.604). **B)** Baujat plot exploring study contribution to overall heterogeneity and influence on bMVT.


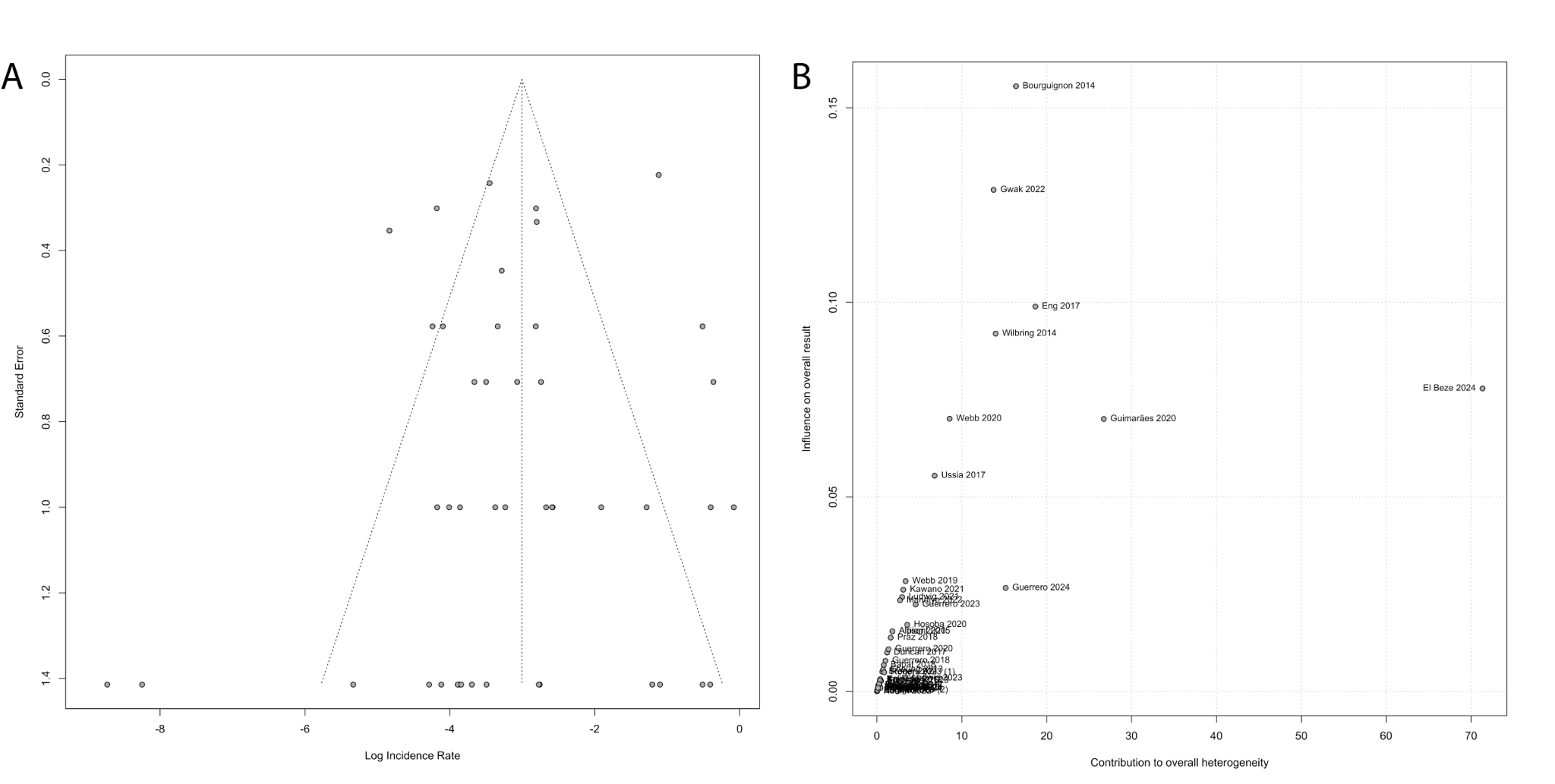


**Influential (leave-one-out) analysis for the overall incidence of bMVT**

| **Study** | **Rate** | **95% CI** | **Tau^2^** | **I^2^** |
| --- | --- | --- | --- | --- |
| Omitting Akodad 2024 | 0.0512 | 0.0318 to 0.0825 | 1.8204 | 81.80% |
| Omitting Alpieri 2020 | 0.049 | 0.0308 to 0.0782 | 1.7528 | 81.90% |
| Omitting Altisent 2015 | 0.049 | 0.0308 to 0.0782 | 1.7528 | 81.90% |
| Omitting Bapat 2018 | 0.0514 | 0.0322 to 0.0822 | 1.7706 | 82.00% |
| Omitting Bourguignon 2014 | 0.055 | 0.0356 to 0.0851 | 1.4435 | 80.70% |
| Omitting Brener 2024 | 0.0513 | 0.0321 to 0.0820 | 1.7755 | 82.00% |
| Omitting Brennan 2012 | 0.0511 | 0.0320 to 0.0817 | 1.7816 | 82.00% |
| Omitting Butnaru 2013 | 0.0501 | 0.0311 to 0.0808 | 1.8332 | 82.00% |
| Omitting Capretti 2016 | 0.0509 | 0.0316 to 0.0819 | 1.8201 | 82.00% |
| Omitting Cheung 2013 | 0.0514 | 0.0321 to 0.0823 | 1.7892 | 82.00% |
| Omitting Conradi 2024 | 0.0506 | 0.0314 to 0.0815 | 1.8341 | 82.00% |
| Omitting da Costa 2020 | 0.0508 | 0.0317 to 0.0815 | 1.8034 | 82.00% |
| Omitting Dahle 2017 | 0.0500 | 0.0312 to 0.0802 | 1.8043 | 82.00% |
| Omitting Duncan 2017 | 0.0493 | 0.0308 to 0.0790 | 1.7837 | 82.00% |
| Omitting El Beze 2024 | 0.0476 | 0.0300 to 0.0754 | 1.6562 | 74.10% |
| Omitting Eleid 2017 | 0.0511 | 0.0318 to 0.0821 | 1.8105 | 82.00% |
| Omitting Eng 2017 | 0.0472 | 0.0300 to 0.0743 | 1.5911 | 80.60% |
| Omitting Gaia 2017 | 0.0510 | 0.0319 to 0.0815 | 1.7850 | 82.00% |
| Omitting Guerrero 2018 | 0.0515 | 0.0322 to 0.0825 | 1.7828 | 82.00% |
| Omitting Guerrero 2020 | 0.0517 | 0.0323 to 0.0827 | 1.7746 | 81.90% |
| Omitting Guerrero 2023 | 0.0522 | 0.0326 to 0.0836 | 1.7628 | 81.70% |
| Omitting Guerrero 2024 | 0.0524 | 0.0327 to 0.0839 | 1.7622 | 80.60% |
| Omitting Guimarães 2020 | 0.0535 | 0.0337 to 0.0849 | 1.6632 | 79.50% |
| Omitting Gwak 2022 | 0.0547 | 0.0352 to 0.0849 | 1.4932 | 81.00% |
| Omitting Hosoba 2020 | 0.0520 | 0.0325 to 0.0833 | 1.7762 | 81.70% |
| Omitting Kalil 2021 | 0.0511 | 0.0320 to 0.0817 | 1.7811 | 82.00% |
| Omitting Kawano 2021 | 0.0486 | 0.0306 to 0.0773 | 1.7245 | 81.80% |
| Omitting Kuohn 2022 | 0.0505 | 0.0314 to 0.0812 | 1.8197 | 82.00% |
| Omitting Long 2018 | 0.0508 | 0.0318 to 0.0813 | 1.7881 | 82.00% |
| Omitting Ludwig 2021 | 0.0487 | 0.0305 to 0.0777 | 1.7471 | 81.80% |
| Omitting Ludwig 2023 | 0.0511 | 0.0317 to 0.0824 | 1.8248 | 81.70% |
| Omitting Malaisrie 2024 | 0.0502 | 0.0312 to 0.0808 | 1.8248 | 82.00% |
| Omitting Mandiye 2022 | 0.0523 | 0.0329 to 0.0831 | 1.7258 | 81.80% |
| Omitting Praz 2018 | 0.0491 | 0.0308 to 0.0783 | 1.7572 | 81.90% |
| Omitting Regueiro 2017 | 0.0500 | 0.0312 to 0.0802 | 1.8045 | 82.00% |
| Omitting Rogers 2023 (1) | 0.0513 | 0.0319 to 0.0824 | 1.8039 | 82.00% |
| Omitting Rogers 2023 (2) | 0.0503 | 0.0314 to 0.0804 | 1.7914 | 82.00% |
| Omitting Schneider 2023 | 0.0511 | 0.0320 to 0.0817 | 1.7821 | 82.00% |
| Omitting Sorajja 2019 | 0.0503 | 0.0314 to 0.0804 | 1.7914 | 82.00% |
| Omitting Ussia 2017 | 0.0479 | 0.0303 to 0.0758 | 1.6674 | 81.60% |
| Omitting Webb 2019 | 0.0486 | 0.0306 to 0.0772 | 1.7187 | 81.80% |
| Omitting Webb 2020 | 0.0462 | 0.0300 to 0.0713 | 1.3955 | 80.00% |
| Omitting Wilbring 2014 | 0.0473 | 0.0300 to 0.0744 | 1.5978 | 81.00% |
| Omitting Wild 2022 | 0.0501 | 0.0312 to 0.0804 | 1.8056 | 82.00% |
| Omitting Ye 2015 | 0.0501 | 0.0312 to 0.0806 | 1.8186 | 82.00% |
| Omitting Yoon 2019 | 0.0501 | 0.0311 to 0.0808 | 1.8342 | 82.00% |
| Omitting Zahr 2023 | 0.0507 | 0.0316 to 0.0813 | 1.8055 | 82.00% |
| **Pooled estimate** | 0.0505 | 0.0318 to 0.0801 | 1.7484 | 81.60% |

1. **Timing of bMVT thrombosis**

Sensitivity analysis of the time of bMVT thrombosis. bMVT events were divided into early (occurring ≤ 90 days after valve replacement) and late (occurring >90 days after valve replacement). **A)** Funnel plot exploring publication bias (Egger’s test, p=0.449). **B)** Baujat plot exploring study contribution to overall heterogeneity.

**
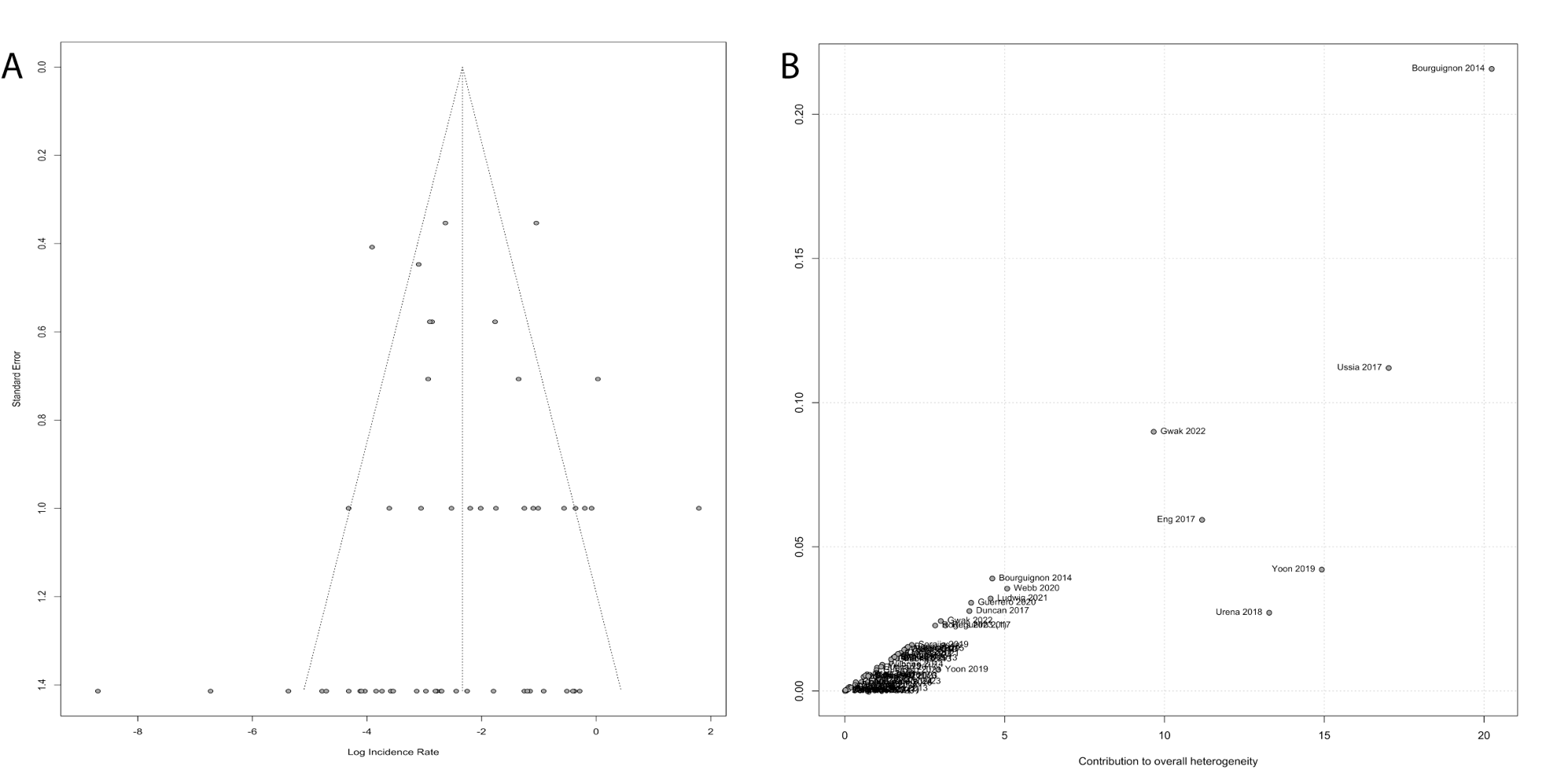
**

**Influential (leave-one-out) analysis for the timing of bMVT**

| **Study** | **Rate** | **95% CI** | **Tau^2^** | **I^2^** |
| --- | --- | --- | --- | --- |
| **Subgroup: Early (**≤ **90 days from valve replacement)** | | | | |
| Omitting Akodad 2023 | 0.0923 | 0.0596 to 0.1428 | 1.6835 | 66.60% |
| Omitting Bourguignon 2014 | 0.0964 | 0.0632 to 0.1472 | 1.5467 | 65.70% |
| Omitting Brener 2024 | 0.0948 | 0.0617 to 0.1455 | 1.6135 | 66.30% |
| Omitting Butnaru 2013 | 0.0947 | 0.0615 to 0.1457 | 1.6298 | 66.30% |
| Omitting Capretti 2016 | 0.0911 | 0.0589 to 0.1409 | 1.6704 | 66.30% |
| Omitting Cheung 2013 | 0.0915 | 0.0593 to 0.1411 | 1.6578 | 66.50% |
| Omitting Dahle 2017 | 0.0903 | 0.0587 to 0.1389 | 1.6273 | 66.20% |
| Omitting Duncan 2017 | 0.0908 | 0.0591 to 0.1396 | 1.6279 | 66.30% |
| Omitting Eleid 2017 | 0.0937 | 0.0608 to 0.1445 | 1.6538 | 66.50% |
| Omitting Eng 2017 | 0.0904 | 0.0587 to 0.1392 | 1.6321 | 66.20% |
| Omitting Gaia 2017 | 0.0918 | 0.0596 to 0.1414 | 1.6468 | 66.50% |
| Omitting Guerrero 2020 | 0.0960 | 0.0627 to 0.1472 | 1.5795 | 65.90% |
| Omitting Guerrero 2023 | 0.0943 | 0.0613 to 0.1449 | 1.6272 | 66.40% |
| Omitting Gwak 2022 | 0.0956 | 0.0625 to 0.1464 | 1.5821 | 66.00% |
| Omitting Kalil 2021 | 0.0929 | 0.0604 to 0.1431 | 1.6482 | 66.60% |
| Omitting Kawano 2021 | 0.0904 | 0.0589 to 0.1389 | 1.6172 | 66.20% |
| Omitting Long 2018 | 0.0924 | 0.0600 to 0.1422 | 1.6499 | 66.60% |
| Omitting Ludwig 2021 | 0.0912 | 0.0593 to 0.1403 | 1.6372 | 66.40% |
| Omitting Malaisrie 2024 | 0.0928 | 0.0601 to 0.1432 | 1.6644 | 66.60% |
| Omitting Praz 2018 | 0.0911 | 0.0592 to 0.1401 | 1.6347 | 66.40% |
| Omitting Regueiro 2017 | 0.0896 | 0.0584 to 0.1375 | 1.5970 | 65.90% |
| Omitting Rogers 2023 (1) | 0.0929 | 0.0604 to 0.1431 | 1.6483 | 66.60% |
| Omitting Rogers 2023 (2) | 0.0930 | 0.0604 to 0.1432 | 1.6475 | 66.60% |
| Omitting Schneider 2023 | 0.0929 | 0.0604 to 0.1431 | 1.6482 | 66.60% |
| Omitting Sorajja 2019 | 0.0901 | 0.0587 to 0.1382 | 1.6039 | 66.10% |
| Omitting Urena 2018 | 0.0892 | 0.0580 to 0.1373 | 1.6074 | 62.90% |
| Omitting Ussia 2017 | 0.0863 | 0.0577 to 0.1291 | 1.2983 | 62.70% |
| Omitting Webb 2019 | 0.0903 | 0.0588 to 0.1387 | 1.6134 | 66.20% |
| Omitting Webb 2020 | 0.0889 | 0.0581 to 0.1359 | 1.5552 | 65.50% |
| Omitting Wilbring 2014 | 0.0907 | 0.0589 to 0.1396 | 1.6398 | 66.30% |
| Omitting Wild 2022 | 0.0919 | 0.0596 to 0.1418 | 1.6630 | 66.50% |
| Omitting Ye 2015 | 0.0904 | 0.0586 to 0.1394 | 1.6455 | 66.10% |
| Omitting Yoon 2019 | 0.0943 | 0.0610 to 0.1458 | 1.6636 | 66.10% |
| **Subgroup: Late (>90 days from valve replacement)** | | | | |
| Omitting Akodad 2023 | 0.0963 | 0.0626 to 0.1480 | 1.5910 | 63.70% |
| Omitting Alpieri 2020 | 0.0902 | 0.0588 to 0.1384 | 1.6080 | 66.20% |
| Omitting Altisent 2015 | 0.0902 | 0.0588 to 0.1384 | 1.6080 | 66.20% |
| Omitting Bapat 2018 | 0.0950 | 0.0619 to 0.1458 | 1.6050 | 66.20% |
| Omitting Brennan 2012 | 0.0935 | 0.0607 to 0.1438 | 1.6424 | 66.50% |
| Omitting Butnaru 2013 | 0.0931 | 0.0601 to 0.1443 | 1.6882 | 66.50% |
| Omitting Capretti 2016 | 0.0936 | 0.0605 to 0.1448 | 1.6732 | 66.50% |
| Omitting Cheung 2013 | 0.0946 | 0.0616 to 0.1453 | 1.6167 | 66.30% |
| Omitting Dahle 2017 | 0.0931 | 0.0605 to 0.1433 | 1.6472 | 66.60% |
| Omitting Duncan 2017 | 0.0893 | 0.0582 to 0.1368 | 1.5808 | 65.70% |
| Omitting Eleid 2017 | 0.0947 | 0.0617 to 0.1454 | 1.6144 | 66.30% |
| Omitting Eng 2017 | 0.0878 | 0.0577 to 0.1336 | 1.4854 | 64.00% |
| Omitting Gaia 2017 | 0.0940 | 0.0611 to 0.1446 | 1.6328 | 66.40% |
| Omitting Guerrero 2023 | 0.0937 | 0.0606 to 0.1449 | 1.6715 | 66.40% |
| Omitting Gwak 2022 | 0.0983 | 0.0650 to 0.1487 | 1.4400 | 64.70% |
| Omitting Kalil 2021 | 0.0941 | 0.0612 to 0.1446 | 1.6318 | 66.40% |
| Omitting Long 2018 | 0.0933 | 0.0606 to 0.1436 | 1.6451 | 66.50% |
| Omitting Ludwig 2021 | 0.0890 | 0.0582 to 0.1363 | 1.5664 | 65.60% |
| Omitting Malaisrie 2024 | 0.0937 | 0.0606 to 0.1447 | 1.6665 | 66.50% |
| Omitting Regueiro 2017 | 0.0911 | 0.0592 to 0.1401 | 1.6359 | 66.40% |
| Omitting Rogers 2023 (1) | 0.0955 | 0.0624 to 0.1463 | 1.5860 | 66.10% |
| Omitting Schneider 2023 | 0.0943 | 0.0614 to 0.1450 | 1.6249 | 66.40% |
| Omitting Ussia 2017 | 0.0902 | 0.0588 to 0.1385 | 1.6091 | 66.20% |
| Omitting Wilbring 2014 | 0.0922 | 0.0597 to 0.1423 | 1.6648 | 66.60% |
| Omitting Ye 2015 | 0.0944 | 0.0614 to 0.1450 | 1.6238 | 66.40% |
| Omitting Yoon 2019 | 0.0966 | 0.0630 to 0.1483 | 1.5728 | 63.60% |

1. **Subclinical versus clinical bMVT**

Sensitivity analysis for the subgroup comparison of subclinical and clinical bMVT. **A)** Funnel plot exploring publication bias (Egger’s test, p=0.465, **B**). Baujat plot exploring study contribution to overall heterogeneity.


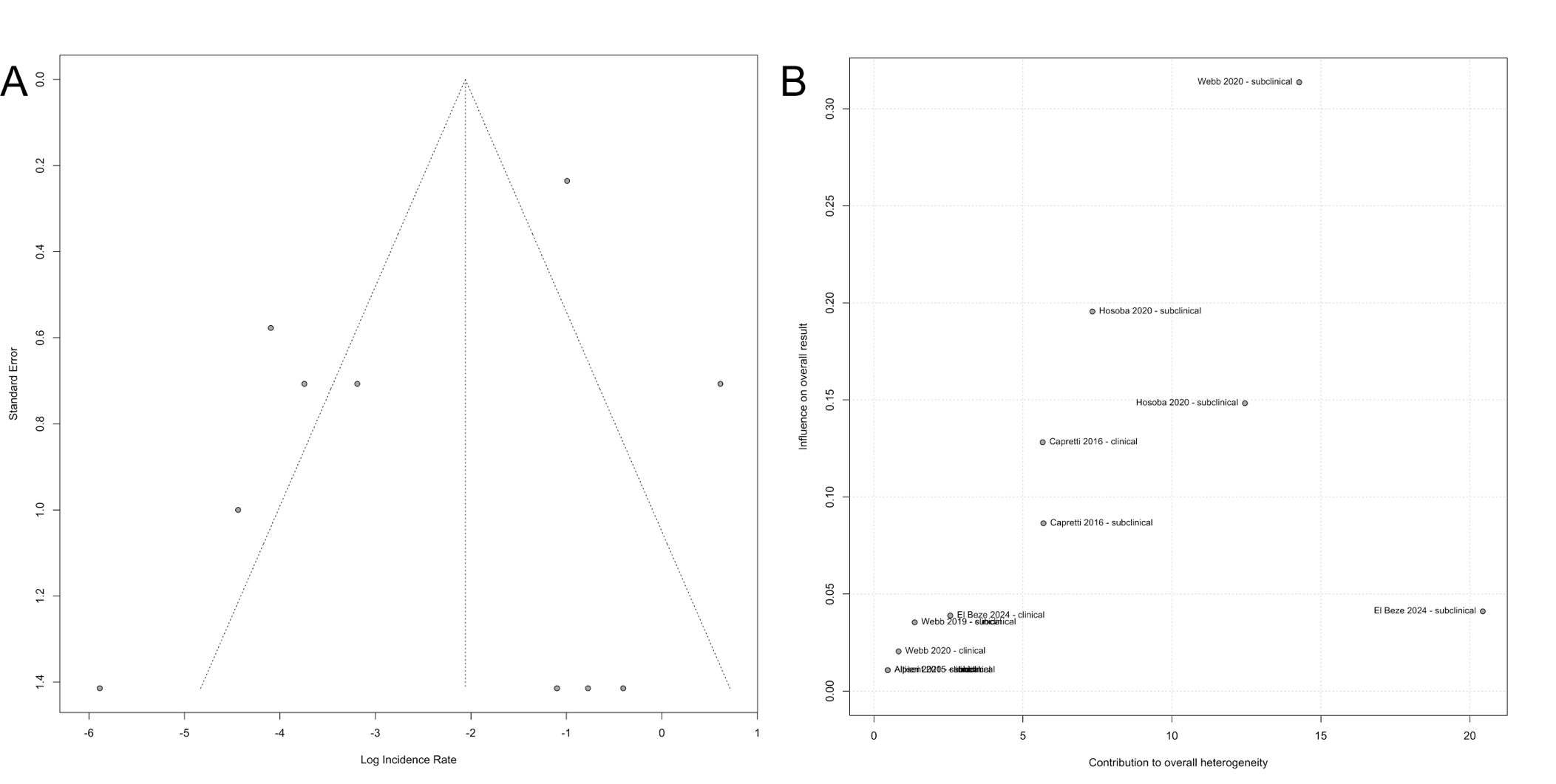


**Influential (leave-one-out) analysis of for the subgroup comparison of subclinical and clinical bMVT**

| **Study** | **Rate** | **95% CI** | **Tau^2^** | **I^2^** |
| --- | --- | --- | --- | --- |
| **Subgroup: clinical** | | | | |
| Omitting Alpieri 2020 | 0.1206 | 0.0409 to 0.3553 | 2.8114 | 82.50% |
| Omitting Altisent 2015 | 0.1206 | 0.0409 to 0.3553 | 2.8114 | 82.50% |
| Omitting Capretti 2016 | 0.1543 | 0.0547 to 0.4356 | 2.4541 | 80.30% |
| Omitting El Beze 2024 | 0.1426 | 0.0475 to 0.4282 | 2.8327 | 81.20% |
| Omitting Webb 2019 | 0.1153 | 0.0397 to 0.3346 | 2.7082 | 82.30% |
| Omitting Webb 2020 | 0.1181 | 0.0403 to 0.3458 | 2.7691 | 82.40% |
| Omitting Hosoba 2020 | 0.1595 | 0.0595 to 0.4280 | 2.187 | 79.90% |
| **Subgroup: subclinical** |  |  |  |  |
| Omitting Alpieri 2020 | 0.1206 | 0.0409 to 0.3553 | 2.8114 | 82.50% |
| Omitting Altisent 2015 | 0.1206 | 0.0409 to 0.3553 | 2.8114 | 82.50% |
| Omitting Capretti 2016 | 0.1500 | 0.0513 to 0.4385 | 2.6452 | 79.80% |
| Omitting El Beze 2024 | 0.1139 | 0.0377 to 0.3441 | 2.8187 | 76.20% |
| Omitting Webb 2019 | 0.1153 | 0.0397 to 0.3346 | 2.7082 | 82.30% |
| Omitting Webb 2020 | 0.0967 | 0.0365 to 0.2560 | 1.9964 | 79.20% |
| Omitting Hosoba 2020 | 0.1568 | 0.0552 to 0.4454 | 2.4306 | 75.60% |

1. **Incidence of bMVT after TMVR versus SMVR**

Sensitivity analysis for the subgroup comparison of bMVT after SMVR and TMVR. **A)** Funnel plot exploring publication bias (Egger’s test, p=0.593). **B)** Baujat plot exploring study contribution to overall heterogeneity.


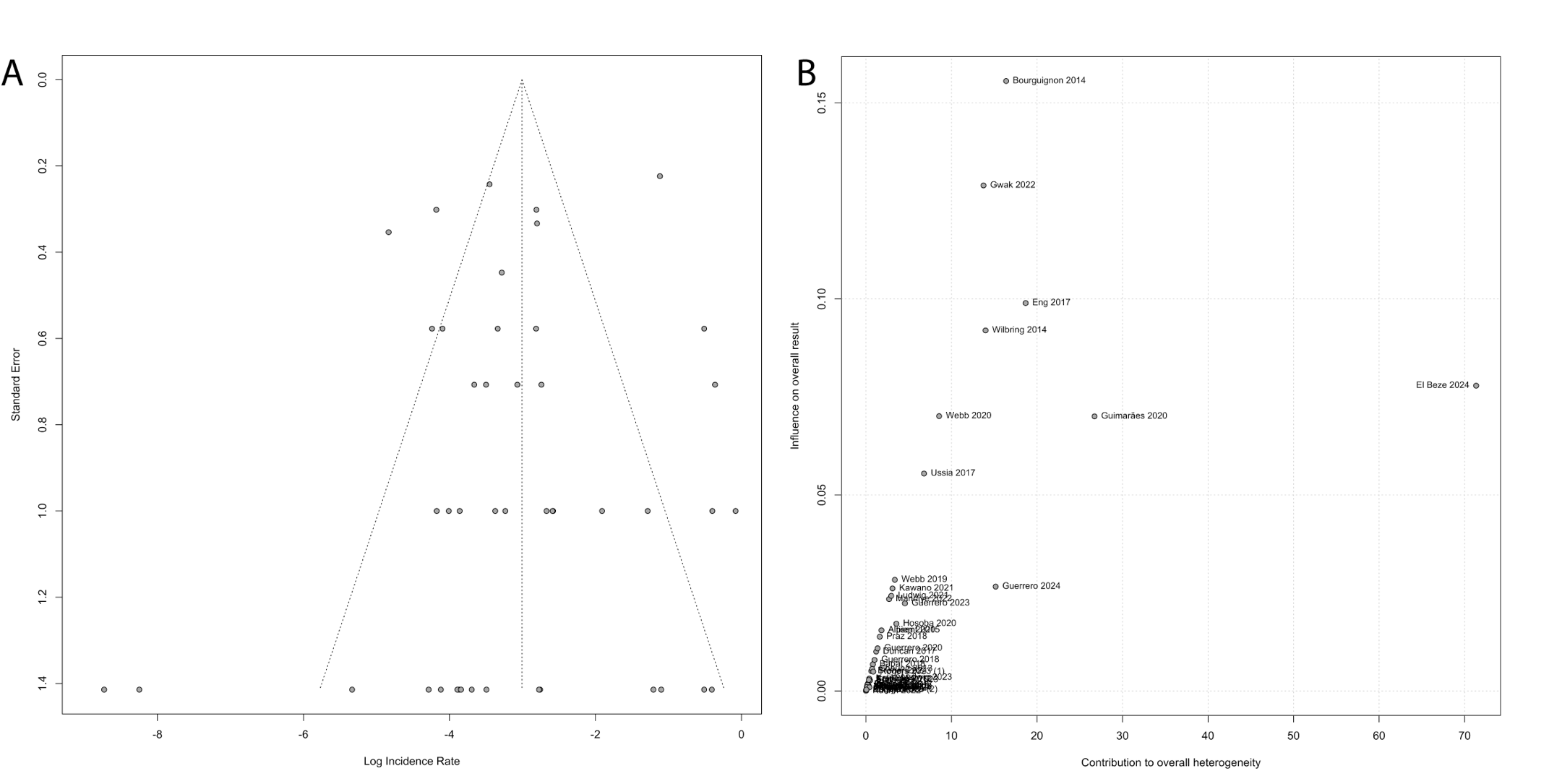


**Influential (leave-one-out) analysis of for the subgroup comparison of bMVT after SMVR and TMVR**

| **Study** | **Rate** | **95% CI** | **Tau^2^** | **I^2^** |
| --- | --- | --- | --- | --- |
| **Subgroup: SMVR** | | | | |
| Omitting Bourguignon 2014 | 0.055 | 0.0356 to 0.0851 | 1.4435 | 80.70% |
| Omitting Brennan 2012 | 0.0511 | 0.0320 to 0.0817 | 1.7815 | 82.00% |
| Omitting Butnaru 2013 | 0.0501 | 0.0311 to 0.0808 | 1.8331 | 82.00% |
| Omitting Guimarães 2020 | 0.0535 | 0.0337 to 0.0849 | 1.6631 | 79.50% |
| Omitting Gwak 2022 | 0.0547 | 0.0352 to 0.0849 | 1.4931 | 81.00% |
| Omitting Hosoba 2020 | 0.052 | 0.0325 to 0.0833 | 1.7761 | 81.70% |
| Omitting Mandiye 2022 | 0.0523 | 0.0329 to 0.0831 | 1.7258 | 81.80% |
| **Subgroup: TMVR** |  |  |  |  |
| Omitting Akodad 2024 | 0.0512 | 0.0318 to 0.0825 | 1.8203 | 81.80% |
| Omitting Alpieri 2020 | 0.0490 | 0.0308 to 0.0782 | 1.7528 | 81.90% |
| Omitting Altisent 2015 | 0.0490 | 0.0308 to 0.0782 | 1.7528 | 81.90% |
| Omitting Bapat 2018 | 0.0514 | 0.0322 to 0.0822 | 1.7705 | 82.00% |
| Omitting Brener 2024 | 0.0513 | 0.0321 to 0.0820 | 1.7754 | 82.00% |
| Omitting Capretti 2016 | 0.0509 | 0.0316 to 0.0819 | 1.8201 | 82.00% |
| Omitting Cheung 2013 | 0.0514 | 0.0321 to 0.0823 | 1.7892 | 82.00% |
| Omitting Conradi 2024 | 0.0506 | 0.0314 to 0.0815 | 1.834 | 82.00% |
| Omitting da Costa 2020 | 0.0508 | 0.0317 to 0.0815 | 1.8033 | 82.00% |
| Omitting Dahle 2017 | 0.0500 | 0.0312 to 0.0802 | 1.8043 | 82.00% |
| Omitting Duncan 2017 | 0.0493 | 0.0308 to 0.0790 | 1.7837 | 82.00% |
| Omitting El Beze 2024 | 0.0476 | 0.0300 to 0.0754 | 1.6561 | 74.10% |
| Omitting Eleid 2017 | 0.0511 | 0.0318 to 0.0821 | 1.8104 | 82.00% |
| Omitting Eng 2017 | 0.0472 | 0.0300 to 0.0743 | 1.591 | 80.60% |
| Omitting Gaia 2017 | 0.0510 | 0.0319 to 0.0815 | 1.785 | 82.00% |
| Omitting Guerrero 2018 | 0.0515 | 0.0322 to 0.0825 | 1.7828 | 82.00% |
| Omitting Guerrero 2020 | 0.0517 | 0.0323 to 0.0827 | 1.7745 | 81.90% |
| Omitting Guerrero 2023 | 0.0522 | 0.0326 to 0.0836 | 1.7627 | 81.70% |
| Omitting Guerrero 2024 | 0.0524 | 0.0327 to 0.0839 | 1.7622 | 80.60% |
| Omitting Kalil 2021 | 0.0511 | 0.0320 to 0.0817 | 1.781 | 82.00% |
| Omitting Kawano 2021 | 0.0486 | 0.0306 to 0.0773 | 1.7245 | 81.80% |
| Omitting Kuohn 2022 | 0.0505 | 0.0314 to 0.0812 | 1.8196 | 82.00% |
| Omitting Long 2018 | 0.0508 | 0.0318 to 0.0813 | 1.788 | 82.00% |
| Omitting Ludwig 2021 | 0.0487 | 0.0305 to 0.0777 | 1.747 | 81.80% |
| Omitting Ludwig 2023 | 0.0511 | 0.0317 to 0.0824 | 1.8248 | 81.70% |
| Omitting Malaisrie 2024 | 0.0502 | 0.0312 to 0.0808 | 1.8248 | 82.00% |
| Omitting Praz 2018 | 0.0491 | 0.0308 to 0.0783 | 1.7571 | 81.90% |
| Omitting Regueiro 2017 | 0.050 | 0.0312 to 0.0802 | 1.8044 | 82.00% |
| Omitting Rogers 2023 (1) | 0.0513 | 0.0319 to 0.0824 | 1.8038 | 82.00% |
| Omitting Rogers 2023 (2) | 0.0503 | 0.0314 to 0.0804 | 1.7913 | 82.00% |
| Omitting Schneider 2023 | 0.0511 | 0.0320 to 0.0817 | 1.7821 | 82.00% |
| Omitting Sorajja 2019 | 0.0503 | 0.0314 to 0.0804 | 1.7914 | 82.00% |
| Omitting Ussia 2017 | 0.0479 | 0.0303 to 0.0758 | 1.6673 | 81.60% |
| Omitting Webb 2019 | 0.0486 | 0.0306 to 0.0772 | 1.7187 | 81.80% |
| Omitting Webb 2020 | 0.0462 | 0.0300 to 0.0713 | 1.3955 | 80.00% |
| Omitting Wilbring 2014 | 0.0473 | 0.0300 to 0.0744 | 1.5977 | 81.00% |
| Omitting Wild 2022 | 0.0501 | 0.0312 to 0.0804 | 1.8056 | 82.00% |
| Omitting Ye 2015 | 0.0501 | 0.0312 to 0.0806 | 1.8185 | 82.00% |
| Omitting Yoon 2019 | 0.0501 | 0.0311 to 0.0808 | 1.8341 | 82.00% |
| Omitting Zahr 2023 | 0.0507 | 0.0316 to 0.0813 | 1.8055 | 82.00% |
| **Pooled estimate** | 0.0505 | 0.0318 to 0.0801 | 1.7484 | 81.60% |

1. **Oral anticoagulation after mitral valve replacement**

Sensitivity analysis for the subgroup comparison of bMVT in patient on oral anticoagulation (OAC) versus without. **A)** Funnel plot exploring publication bias (Egger’s test, p=0.388). **B)** Baujat plot exploring study contribution to overall heterogeneity.


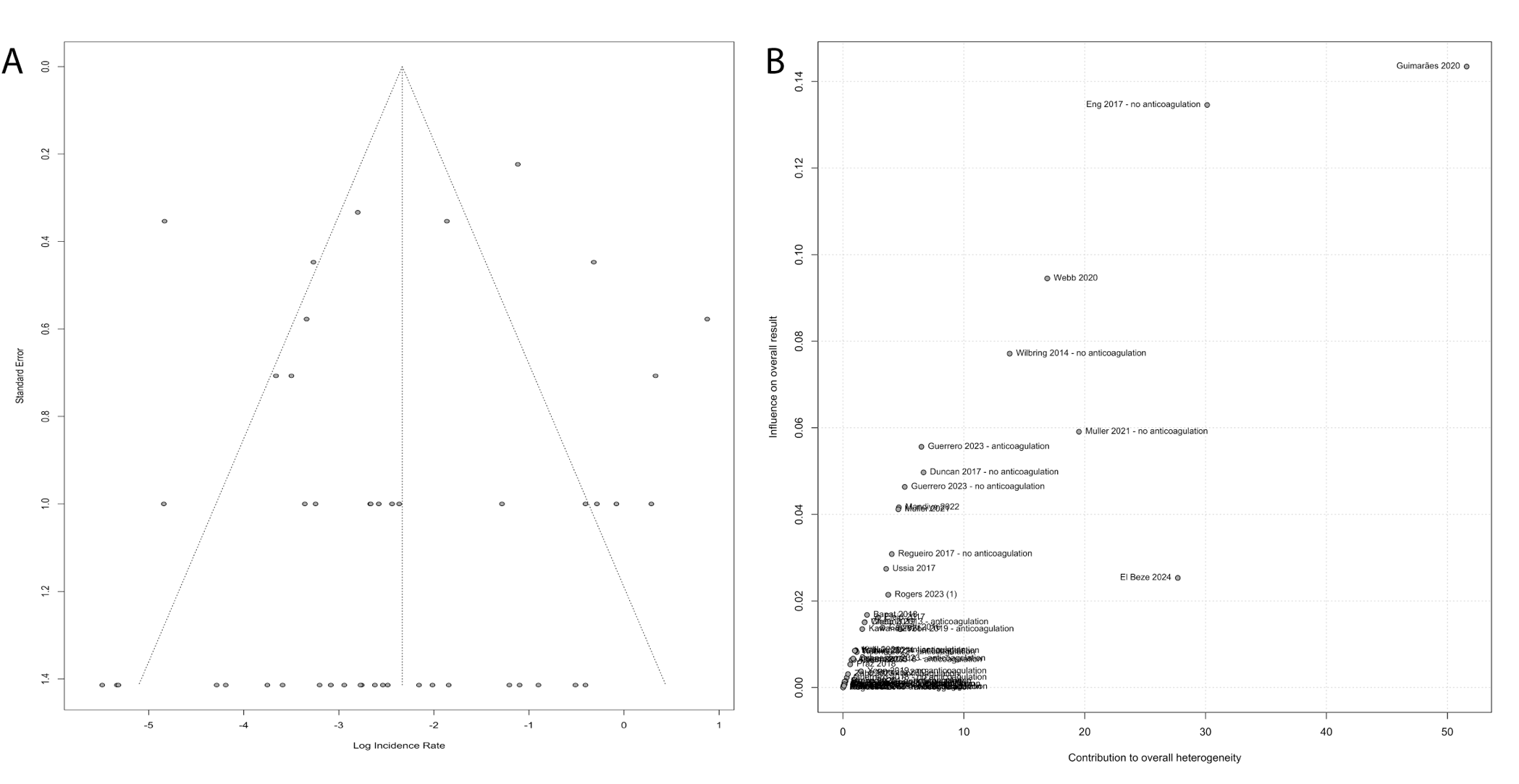


**Influential analysis of for the subgroup comparison of bMVT in patients on oral anticoagulation (OAC) versus without**

| **Study** | **Rate** | **95% CI** | **Tau^2^** | **I^2^** |
| --- | --- | --- | --- | --- |
| **Subgroup: No OAC** | | | | |
| Omitting Akodad 2023 - no OAC | 0.0901 | 0.0570 to 0.1423 | 1.738 | 78.70% |
| Omitting Brennan 2012 - no OAC | 0.0924 | 0.0587 to 0.1454 | 1.7255 | 79.00% |
| Omitting Cheung 2013 - no OAC | 0.0927 | 0.0588 to 0.1462 | 1.7361 | 78.90% |
| Omitting Duncan 2017 - no OAC | 0.0877 | 0.0562 to 0.1369 | 1.6239 | 78.40% |
| Omitting Eng 2017 - no OAC | 0.0850 | 0.0553 to 0.1306 | 1.4313 | 76.20% |
| Omitting Guerrero 2018 - no OAC | 0.0931 | 0.0592 to 0.1465 | 1.7206 | 78.90% |
| Omitting Guerrero 2023 - no OAC | 0.0965 | 0.0619 to 0.1507 | 1.6372 | 78.50% |
| Omitting Hosoba 2020 - No OAC | 0.0921 | 0.0584 to 0.1452 | 1.7381 | 79.00% |
| Omitting Kalil 2021 - no OAC | 0.0915 | 0.0582 to 0.1439 | 1.7241 | 79.00% |
| Omitting Kuohn 2022 - no OAC | 0.0927 | 0.0588 to 0.1461 | 1.7361 | 78.90% |
| Omitting Conradi 2024 - no OAC | 0.0866 | 0.0556 to 0.1349 | 1.5777 | 76.40% |
| Omitting Regueiro 2017 - no OAC | 0.0886 | 0.0565 to 0.1388 | 1.668 | 78.60% |
| Omitting Schneider 2023 - no OAC | 0.0919 | 0.0584 to 0.1446 | 1.7258 | 79.00% |
| Omitting Ussia 2017 | 0.0888 | 0.0566 to 0.1392 | 1.6759 | 78.70% |
| Omitting Wilbring 2014 - no OAC | 0.0866 | 0.0557 to 0.1346 | 1.5645 | 77.80% |
| Omitting Yoon 2019 - no OAC | 0.0908 | 0.0574 to 0.1436 | 1.7503 | 78.90% |
| Omitting Zahr 2023 - no OAC | 0.0923 | 0.0585 to 0.1455 | 1.7379 | 79.00% |
| **Subgroup: OAC** |  |  |  |  |
| Omitting Akodad 2023 - OAC | 0.0979 | 0.0630 to 0.1520 | 1.5831 | 78.20% |
| Omitting Alpieri 2020 | 0.0905 | 0.0576 to 0.1423 | 1.7131 | 78.90% |
| Omitting Altisent 2015 | 0.0905 | 0.0576 to 0.1423 | 1.7131 | 78.90% |
| Omitting Bapat 2018 | 0.0948 | 0.0605 to 0.1487 | 1.6910 | 78.80% |
| Omitting Brennan 2012 - OAC | 0.0930 | 0.0591 to 0.1462 | 1.7224 | 78.90% |
| Omitting Butnaru 2013 | 0.0933 | 0.0590 to 0.1476 | 1.7516 | 78.60% |
| Omitting Capretti 2016 | 0.0946 | 0.0600 to 0.1491 | 1.7223 | 78.60% |
| Omitting Cheung 2013 - OAC | 0.0947 | 0.0603 to 0.1485 | 1.6943 | 78.80% |
| Omitting Dahle 2017 | 0.0925 | 0.0587 to 0.1459 | 1.737 | 78.90% |
| Omitting Duncan 2017 - OAC | 0.0923 | 0.0587 to 0.1453 | 1.7257 | 79.00% |
| Omitting El Beze 2024 | 0.0887 | 0.0563 to 0.1398 | 1.6995 | 76.30% |
| Omitting Eleid 2017 | 0.0948 | 0.0602 to 0.1492 | 1.7132 | 78.60% |
| Omitting Eng 2017 - OAC | 0.0917 | 0.0583 to 0.1443 | 1.7253 | 79.00% |
| Omitting Guerrero 2018 - OAC | 0.0938 | 0.0596 to 0.1477 | 1.7237 | 78.90% |
| Omitting Guerrero 2023 - OAC | 0.0970 | 0.0621 to 0.1515 | 1.6314 | 78.30% |
| Omitting Guimarães 2020 | 0.0998 | 0.0645 to 0.1546 | 1.5125 | 71.80% |
| Omitting Hosoba 2020 - OAC | 0.0972 | 0.0621 to 0.1519 | 1.6330 | 78.00% |
| Omitting Kalil 2021 - OAC | 0.0941 | 0.0599 to 0.1477 | 1.7072 | 78.90% |
| Omitting Kawano 2021 | 0.0898 | 0.0572 to 0.1410 | 1.6975 | 78.80% |
| Omitting Kuohn 2022 - OAC | 0.0940 | 0.0597 to 0.1480 | 1.7199 | 78.80% |
| Omitting Ludwig 2021 | 0.0902 | 0.0573 to 0.1421 | 1.7197 | 78.90% |
| Omitting Malaisrie 2024 | 0.0932 | 0.0590 to 0.1473 | 1.7457 | 78.80% |
| Omitting Mandiye 2022 | 0.0963 | 0.0617 to 0.1505 | 1.6454 | 78.50% |
| Omitting Conradi 2024 - OAC | 0.0925 | 0.0588 to 0.1455 | 1.7251 | 79.00% |
| Omitting Praz 2018 | 0.0907 | 0.0577 to 0.1425 | 1.7153 | 78.90% |
| Omitting Regueiro 2017 - OAC | 0.0921 | 0.0584 to 0.1453 | 1.7381 | 79.00% |
| Omitting Rogers 2023 (1) | 0.0952 | 0.0605 to 0.1497 | 1.7028 | 78.60% |
| Omitting Rogers 2023 (2) | 0.0927 | 0.0589 to 0.1458 | 1.7242 | 78.90% |
| Omitting Schneider 2023 - OAC | 0.0938 | 0.0597 to 0.1474 | 1.7112 | 78.90% |
| Omitting Sorajja 2019 | 0.0927 | 0.0589 to 0.1459 | 1.7241 | 78.90% |
| Omitting Webb 2019 | 0.0897 | 0.0572 to 0.1407 | 1.6941 | 78.80% |
| Omitting Webb 2020 | 0.0883 | 0.0564 to 0.1381 | 1.6534 | 78.60% |
| Omitting Wilbring 2014 - OAC | 0.0903 | 0.0575 to 0.1418 | 1.7085 | 78.90% |
| Omitting Wild 2022 | 0.0925 | 0.0587 to 0.1459 | 1.737 | 78.90% |
| Omitting Yoon 2019 - OAC | 0.0945 | 0.0599 to 0.1492 | 1.7269 | 78.40% |
| Omitting Zahr 2023 - OAC | 0.0933 | 0.0593 to 0.1467 | 1.7188 | 78.90% |

1. **Oral anticoagulation after bioprosthetic mitral valve replacement: VKAs compared to DOACs**

Sensitivity analysis for the subgroup comparison of bMVT in patient on VKAs versus DOACs. **A)** Funnel plot exploring publication bias (Egger’s test, p=0.796). **B)** Baujat plot exploring study contribution to overall heterogeneity.


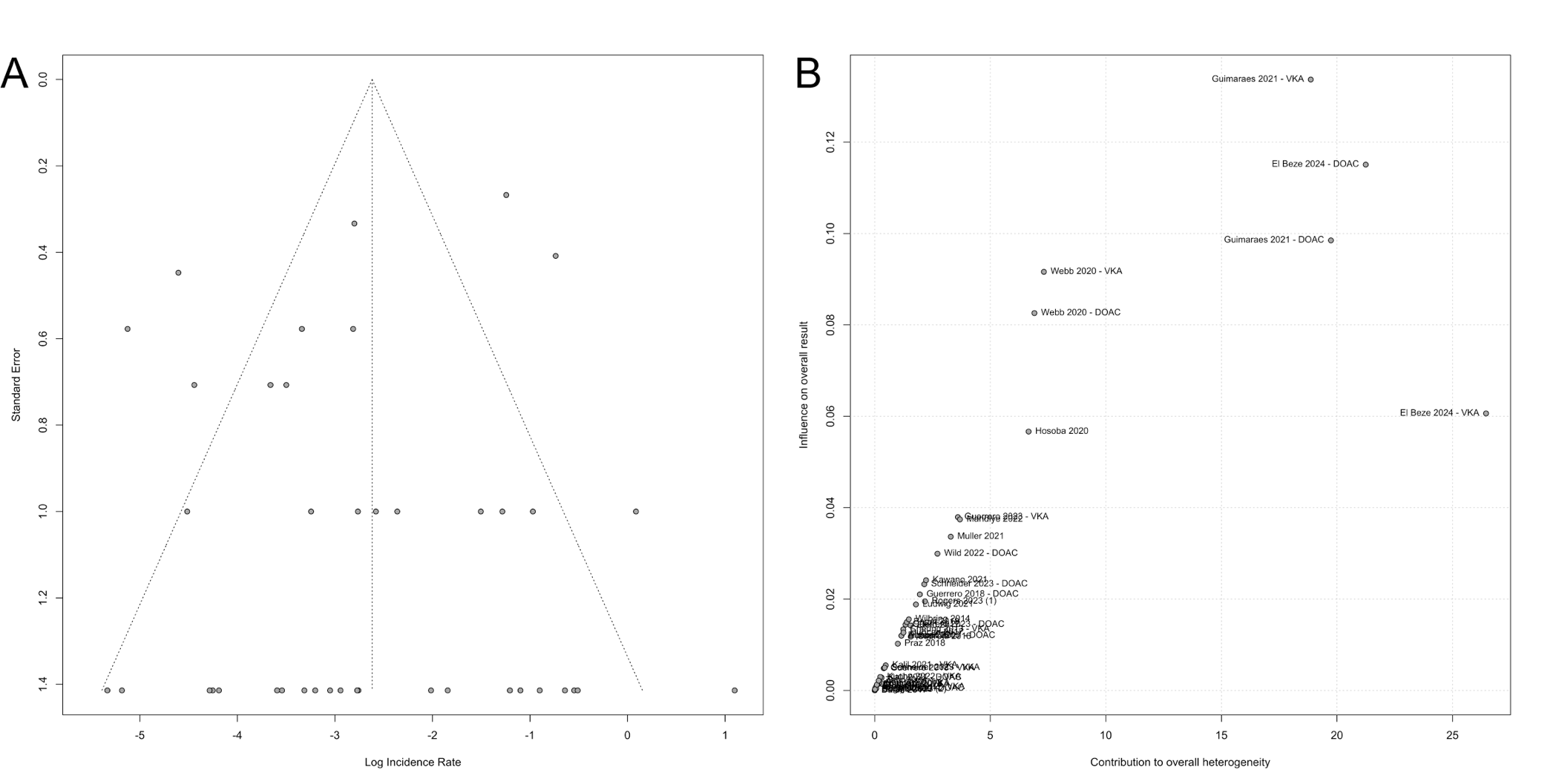


**Influential analysis of for the subgroup comparison of bMVT in patients taking VKAs versus DOACs**

| **Study** | **Rate** | **95% CI** | **Tau^2^** | **I^2^** |
| --- | --- | --- | --- | --- |
| **Subgroup: DOAC** | | | | |
| Omitting Duncan 2017 - DOAC | 0.071 | 0.0438 to 0.1150 | 1.3856 | 71.90% |
| Omitting El Beze 2024 - DOAC | 0.0672 | 0.0419 to 0.1078 | 1.243 | 67.60% |
| Omitting Guerrero 2018 - DOAC | 0.0704 | 0.0435 to 0.1138 | 1.3673 | 71.80% |
| Omitting Guerrero 2023 - DOAC | 0.0751 | 0.0464 to 0.1217 | 1.3884 | 71.80% |
| Omitting Guimaraes 2021 - DOAC | 0.0787 | 0.0490 to 0.1262 | 1.2505 | 66.70% |
| Omitting Kalil 2021 - DOAC | 0.0720 | 0.0444 to 0.1168 | 1.4061 | 72.10% |
| Omitting Kuohn 2022 - DOAC | 0.0733 | 0.0450 to 0.1195 | 1.4298 | 72.10% |
| Omitting Schneider 2023 - DOAC | 0.0703 | 0.0435 to 0.1135 | 1.363 | 71.70% |
| Omitting Webb 2020 - DOAC | 0.0681 | 0.0426 to 0.1088 | 1.262 | 70.80% |
| Omitting Wild 2022 - DOAC | 0.0699 | 0.0432 to 0.1131 | 1.3634 | 71.60% |
| **Subgroup: VKA** | | | | |
| Omitting Alpieri 2020 | 0.0710 | 0.0438 to 0.1150 | 1.3856 | 71.90% |
| Omitting Altisent 2015 | 0.0710 | 0.0438 to 0.1150 | 1.3856 | 71.90% |
| Omitting Bapat 2018 | 0.0752 | 0.0464 to 0.1217 | 1.3873 | 71.80% |
| Omitting Brennan 2012 - VKA | 0.0734 | 0.0452 to 0.1193 | 1.4139 | 72.10% |
| Omitting Butnaru 2013 | 0.0736 | 0.0449 to 0.1208 | 1.4587 | 71.90% |
| Omitting Capretti 2016 | 0.0749 | 0.0459 to 0.1224 | 1.4296 | 71.70% |
| Omitting Cheung 2013 - VKA | 0.0750 | 0.0463 to 0.1216 | 1.3905 | 71.80% |
| Omitting Dahle 2017 | 0.0730 | 0.0448 to 0.1189 | 1.4297 | 72.10% |
| Omitting Duncan 2017 | 0.0709 | 0.0437 to 0.1152 | 1.3982 | 71.90% |
| Omitting El Beze 2024 - VKA | 0.0686 | 0.0424 to 0.1112 | 1.3337 | 65.60% |
| Omitting Eleid 2017 | 0.0751 | 0.0461 to 0.1225 | 1.4183 | 71.70% |
| Omitting Eng 2017 - VKA | 0.0722 | 0.0445 to 0.1172 | 1.4090 | 72.10% |
| Omitting Guerrero 2018 - VKA | 0.0742 | 0.0456 to 0.1208 | 1.4228 | 72.00% |
| Omitting Guerrero 2023 - VKA | 0.0765 | 0.0473 to 0.1237 | 1.3547 | 71.30% |
| Omitting Guimaraes 2021 - VKA | 0.0795 | 0.0500 to 0.1264 | 1.1724 | 67.30% |
| Omitting Hosoba 2020 | 0.0773 | 0.0479 to 0.1249 | 1.3281 | 70.60% |
| Omitting Kalil 2021 - VKA | 0.0743 | 0.0458 to 0.1205 | 1.4059 | 72.00% |
| Omitting Kawano 2021 | 0.0702 | 0.0435 to 0.1134 | 1.3613 | 71.70% |
| Omitting Kuohn 2022 - VKA | 0.0739 | 0.0455 to 0.1200 | 1.4105 | 72.00% |
| Omitting Ludwig 2021 | 0.0705 | 0.0435 to 0.1144 | 1.3853 | 71.80% |
| Omitting Malaisrie 2024 | 0.0736 | 0.0450 to 0.1204 | 1.4487 | 72.00% |
| Omitting Mandiye 2022 | 0.0764 | 0.0474 to 0.1232 | 1.3405 | 71.30% |
| Omitting Muller 2021 | 0.0763 | 0.0473 to 0.1230 | 1.3485 | 71.40% |
| Omitting Praz 2018 | 0.0711 | 0.0439 to 0.1152 | 1.3893 | 71.90% |
| Omitting Regueiro 2017 | 0.0726 | 0.0445 to 0.1182 | 1.4276 | 72.10% |
| Omitting Rogers 2023 (1) | 0.0755 | 0.0464 to 0.1230 | 1.4075 | 71.60% |
| Omitting Rogers 2023 (2) | 0.0732 | 0.0451 to 0.1189 | 1.4145 | 72.10% |
| Omitting Schneider 2023 - VKA | 0.0742 | 0.0457 to 0.1204 | 1.4069 | 72.00% |
| Omitting Sorajja 2019 | 0.0732 | 0.0451 to 0.1189 | 1.4145 | 72.10% |
| Omitting Webb 2020 - VKA | 0.0678 | 0.0424 to 0.1085 | 1.2590 | 70.70% |
| Omitting Wild 2022 - VKA | 0.0736 | 0.0453 to 0.1195 | 1.4132 | 72.10% |
| Omitting Zahr 2023 | 0.0738 | 0.0454 to 0.1198 | 1.4118 | 72.10% |
