# Supplementary material for: Valve thrombosis and antithrombotic therapy after bioprosthetic mitral valve replacement: a systematic review and meta-analysis
Source: Eur Heart J Cardiovasc Pharmacother. 2025 Feb 4;11(3):251–63. doi: 10.1093/ehjcvp/pvaf005 (PMC12046575; doi:10.1093/ehjcvp/pvaf005)
Supplement: pvaf005_Supplemental_Files [file pvaf005_supplemental_files.zip › Supplementary Material 1_MOOSE Reporting Checklist.docx]

**Supplementary Figure 1: MOOSE Reporting Checklist**

**Reporting of background should include**
Problem definition ***Introduction***
Description of study outcome(s) ***Methods: Outcome***
Type of exposure or intervention used ***Methods: Inclusion/Exclusion Criteria***
Type of study designs used ***Table 1***
Study population ***Supplementary Material 3***

**Reporting of search strategy should include**Qualifications of searchers (eg, librarians and investigators) ***Supplementary Materials 2***
Search strategy, including time period included in the synthesis and keywords ***Supplementary Materials 2***
Effort to include all available studies, including contact with authors ***Not done***
Databases and registries searched ***Supplementary Materials 2***
Search software used, name and version, including special features used (eg, explosion) ***Supplementary Materials 2***
Use of hand searching (eg, reference lists of obtained articles) ***Figure 1***
List of citations located and those excluded, including justification ***Figure 1***Method of handling abstracts and unpublished studies ***Methods***
Description of any contact with authors ***Publication authors were not contacted.***
**Reporting of methods should include**
Description of relevance or appropriateness of studies assembled for assessing the hypothesis to be tested ***Methods, Results, Limitations***
Rationale for the selection and coding of data (eg, sound clinical principles or convenience) ***Methods: Inclusion/Exclusion Criteria***
Documentation of how data were classified and coded (eg, multiple raters, blinding, and interrater reliability) ***Methods: Statistical analysis***
Assessment of confounding (eg, comparability of cases and controls in studies where appropriate) ***Supplementary Materials 4***
Assessment of study quality, including blinding of quality assessors; stratification or regression on possible predictors of study results ***Methods: Inclusion/Exclusion Criteria (no further quality assessment)***
Assessment of heterogeneity ***Table 2***
Description of statistical methods (eg, complete description of fixed or random effects models, justification of whether the chosen models account for predictors of study results, dose-response models, or cumulative meta-analysis) in sufficient detail to be replicated ***Methods: Statistical analysis***
Provision of appropriate tables and graphics ***Figures 1-5, Table 1-2, Supplementary Materials***

**Reporting of results should include**
Graphic summarizing individual study estimates and overall estimate ***Figure 2-5***
Table giving descriptive information for each study included ***Table 1, Supplementary Materials 3***
Results of sensitivity testing (eg, subgroup analysis) ***Supplementary Materials 7***
Indication of statistical uncertainty of findings ***Results, Table 2***

**Reporting of discussion should include**
Quantitative assessment of bias (eg, publication bias) ***Supplementary Materials 7***
Justification for exclusion (eg, exclusion of non–English-language citations) ***Methods: Inclusion/Exclusion Criteria***
Assessment of quality of included studies ***Methods: Inclusion/Exclusion Criteria***

**Reporting of conclusions should include**
Consideration of alternative explanations for observed results ***Discussion***
Generalization of the conclusions (ie, appropriate for the data presented and within the domain of the literature review) ***Discussion, Conclusions***Guidelines for future research ***Discussion***
Disclosure of funding source ***Cover page***
