# Supplementary material for: Valve thrombosis and antithrombotic therapy after bioprosthetic mitral valve replacement: a systematic review and meta-analysis
Source: Eur Heart J Cardiovasc Pharmacother. 2025 Feb 4;11(3):251–63. doi: 10.1093/ehjcvp/pvaf005 (PMC12046575; doi:10.1093/ehjcvp/pvaf005)
Supplement: pvaf005_Supplemental_Files [file pvaf005_supplemental_files.zip › Supplementary Material 2_Search Strategy_MZ.docx]

**Supplementary Material 2: EMBASE, MEDLINE and Cochrane Library search strategies**

A systematic search of literature was performed by an academic librarian (M.H.) in conjunction with the first author.

**Embase 1974 to present**

Date of the last search: 25^th^ August 2024

Search link: <https://ovidsp.ovid.com/ovidweb.cgi?T=JS&NEWS=N&PAGE=main&SHAREDSEARCHID=Pm6cfou1g3WGBa5qWb3yVZuAJuiIoeeKRJcCba442D05lp3e3MWyj9IE3FBjCAVh>

Search strategy:

1 warfarin/ 110760

2 (warfarin or acetonylbenzylhydroxycoumarin or adoisine or aldocumar or antrombin-k or athrombin or athrombin-k or athrombine-k or athrombinek or befarin or carfin or circuvit or "compound 42" or coumadan or coumadin or coumadine or coumafene or coumaphene dagonal or farin or jantoven or kumatox or maforan or marevan or orfarin or panwarfarin or panwarfin or prothromadin or simarc-2 or "sodium warfarinum" or sofarin or tintorane or uniwarfin or wafarin or waran or "warf compound 42" or warfar or warfarine or "warfarinum sodium" or "warfil 5" or warfilone or warnerin).ti,ab,kf. 50842

3 1 or 2 116143

4 antithrombocytic agent/ or clopidogrel/ or acetylsalicylic acid/ or ticagrelor/ or prasugrel/ or dipyridamole/ 333587

5 ("antithrombocytic agent" or "antiplatelet agent" or "antiplatelet drug" or (platelet adj3 inhibitor*) or "thrombocyte aggregation inhibit*" or clopidogrel or aspirin or "acetylsalicylic acid" or ticagrelor or prasugrel or dipyridamole).ti,ab,kf. 134492

6 4 or 5 350646

7 apixaban/ or dabigatran/ or edoxaban/ or rivaroxaban/ 46652

8 ("new oral anticoagulant*" or "new oral anti-coagulant*" or "novel oral anticoagulant*" or "novel oral anti-coagulant*" or NOAC* or DOAC* or (direct adj3 oral anticoagulant) or (direct adj3 oral anti-coagulant) or "factor Xa inhibitor*" or xaban* or "non-vitamin K oral anticoagulant*" or "non-vitamin K oral anti-coagulant*" or dabigatran or Pradaxa or rivaroxaban or Xarelto or apixaban or Eliquis or edoxaban or Lixiana).ti,ab,kf. 41382

9 7 or 8 58897

10 3 or 6 or 9 458667

11 prosthetic valve thrombosis/ 2304

12 ((valve or leaflet) adj3 (thromb* or thicken* or motion)).ti,ab,kf. 7085

13 ("hypoattenuated leaflet thickening" or "reduced leaflet motion").ti,ab,kf. 172

14 11 or 12 or 13 8144

15 10 and 14 2098

16 limit 15 to yr="2000 - 2024" 1915

**Medline (Ovid MEDLINE® Epub Ahead of Print, In-Process & Other Non-Indexed Citations, Ovid MEDLINE® Daily and Ovid MEDLINE®) 1946 to present**

Date of the search: 25^th^ August 2024

Search link: <https://ovidsp.ovid.com/ovidweb.cgi?T=JS&NEWS=N&PAGE=main&SHAREDSEARCHID=7P0bGluATEnqKT9MjTJ6I4Uanh9VDwcByF4evcqw26Rilhc7qQi5VFDgJbZmOGAbk>

Search strategy:

1 Warfarin/ 21866

2 (warfarin or acetonylbenzylhydroxycoumarin or adoisine or aldocumar or antrombin-k or athrombin or athrombin-k or athrombine-k or athrombinek or befarin or carfin or circuvit or "compound 42" or coumadan or coumadin or coumadine or coumafene or coumaphene dagonal or farin or jantoven or kumatox or maforan or marevan or orfarin or panwarfarin or panwarfin or prothromadin or simarc-2 or "sodium warfarinum" or sofarin or tintorane or uniwarfin or wafarin or waran or "warf compound 42" or warfar or warfarine or "warfarinum sodium" or "warfil 5" or warfilone or warnerin).ti,ab,kf. 29534

3 1 or 2 35457

4 Platelet Aggregation Inhibitors/ or Aspirin/ or Clopidogrel/ or Dipyridamole/ or Prasugrel Hydrochloride/ or Ticagrelor/ 86888

5 ("antithrombocytic agent" or "antiplatelet agent" or "antiplatelet drug" or (platelet adj3 inhibitor*) or "thrombocyte aggregation inhibit*" or clopidogrel or aspirin or "acetylsalicylic acid" or ticagrelor or prasugrel or dipyridamole).ti,ab,kf. 86894

6 4 or 5 121608

7 Dabigatran/ or Rivaroxaban/ or Factor Xa Inhibitors/ 11482

8 ("new oral anticoagulant*" or "new oral anti-coagulant*" or "novel oral anticoagulant*" or "novel oral anti-coagulant*" or NOAC* or DOAC* or (direct adj3 oral anticoagulant) or (direct adj3 oral anti-coagulant) or "factor Xa inhibitor*" or xaban* or "non-vitamin K oral anticoagulant*" or "non-vitamin K oral anti-coagulant*" or dabigatran or Pradaxa or rivaroxaban or Xarelto or apixaban or Eliquis or edoxaban or Lixiana).ti,ab,kf. 21206

9 7 or 8 23883

10 3 or 6 or 9 166496

11 Heart Valve Prosthesis/ and Thrombosis/ 2302

12 ((valve or leaflet) adj3 (thromb* or thicken* or motion)).ti,ab,kf. 4758

13 ("hypoattenuated leaflet thickening" or "reduced leaflet motion").ti,ab,kf. 108

14 11 or 12 or 13 6025

15 10 and 14 764

16 limit 15 to yr="2000 - 2024" 550

**Cochrane Library**

Date of the search: 25^th^ August 2024

Search link:

<https://www.cochranelibrary.com/advanced-search/search-manager?search=7536056>

Search strategy:

#1 [mh ^Warfarin] 2336

#2 (warfarin OR acetonylbenzylhydroxycoumarin OR adoisine OR aldocumar OR antrombin-k OR athrombin OR athrombin-k OR athrombine-k OR athrombinek OR befarin OR carfin OR circuvit OR "compound 42" OR coumadan OR coumadin OR coumadine OR coumafene OR "coumaphene dagonal" OR farin OR jantoven OR kumatox OR maforan OR marevan OR orfarin OR panwarfarin OR panwarfin OR prothromadin OR simarc-2 OR "sodium warfarinum" OR sofarin OR tintorane OR uniwarfin OR wafarin OR waran OR "warf compound 42" OR warfar OR warfarine OR "warfarinum sodium" OR "warfil 5" OR warfilone OR warnerin):ti,ab,kw 5651

#3 #1 OR #2 5651

#4 [mh ^"Platelet Aggregation Inhibitors"] OR [mh ^Aspirin] OR [mh ^Clopidogrel] OR [mh ^Dipyridamole] OR [mh ^"Prasugrel Hydrochloride"] OR [mh ^Ticagrelor] 12582

#5 ("antithrombocytic agent" OR "antiplatelet agent" OR "antiplatelet drug" OR (platelet NEAR/3 inhibitor*) OR ("thrombocyte aggregation" NEXT inhibit*) OR clopidogrel OR aspirin OR "acetylsalicylic acid" OR ticagrelor OR prasugrel OR dipyridamole):ti,ab,kw 25073

#6 #4 OR #5 25073

#7 [mh ^Dabigatran] OR [mh ^Rivaroxaban] OR [mh ^"Factor Xa Inhibitors"] 1902

#8 (("new oral" NEXT anticoagulant*) OR ("new oral" NEXT anti-coagulant*) OR ("novel oral" NEXT anticoagulant*) OR ("novel oral" NEXT anti-coagulant*) OR NOAC* OR DOAC* OR (direct NEAR/3 "oral anticoagulant") OR (direct NEAR/3 "oral anti-coagulant") OR ("factor Xa" NEXT inhibitor*) OR xaban* OR ("non-vitamin K oral" NEXT anticoagulant*) OR ("non-vitamin K oral" NEXT anti-coagulant*) OR dabigatran OR Pradaxa OR rivaroxaban OR Xarelto OR apixaban OR Eliquis OR edoxaban OR Lixiana):ti,ab,kw 5537

#9 #7 OR #8 5537

#10 #3 OR #6 OR #9 32191

#11 [mh ^"Heart Valve Prosthesis"] AND [mh ^Thrombosis] 34

#12 (valve:ti,ab,kw OR leaflet:ti,ab,kw) NEAR/3 (thromb*:ti,ab,kw OR thicken*:ti,ab,kw OR motion:ti,ab,kw) 311

#13 ("hypoattenuated leaflet thickening":ti,ab,kw OR "reduced leaflet motion":ti,ab,kw) 30

#14 #11 OR #12 OR #13 321

#15 #10 AND #14 with Cochrane Library publication date Between Jan 2000 and Aug 2024 135
