# Supplementary material for: Valve thrombosis and antithrombotic therapy after bioprosthetic mitral valve replacement: a systematic review and meta-analysis
Source: Eur Heart J Cardiovasc Pharmacother. 2025 Feb 4;11(3):251–63. doi: 10.1093/ehjcvp/pvaf005 (PMC12046575; doi:10.1093/ehjcvp/pvaf005)
Supplement: pvaf005_Supplemental_Files [file pvaf005_supplemental_files.zip › Supplementary Material 3_Patient baseline characteristics .docx]

**Supplementary Table 1: Patient baseline characteristics in included studies.** Baseline characteristics of patients from included studies were compared between the following subgroups: surgical versus transcatheter mitral valve replacement (SMVR and TMVR, respectively), oral anticoagulation (OAC) versus no oral anticoagulation (no OAC), and VKAs versus DOACs. N: number of patients, CAD: coronary artery disease, AF: atrial fibrillation, TIA: transient ischemic attack, CKD: chronic kidney disease, Ts: transseptal, Tap: transapical, Tat: transatrial, LV EF: left ventricular ejection fraction (mean ± SD).

| Study | N | Age (y) | Male | Diabetes | CAD | AF | Stroke or TIA | CKD^*^ | STS PROM score | Approach | LV EF |
| --- | --- | --- | --- | --- | --- | --- | --- | --- | --- | --- | --- |
| Akodad 2023 | 119 | 76.8 ± 10.2 | 37.0% (44/119) | 23.5% (28/119) | 52.1% (62/119) | 67.2% (80/119) | 25.5% (30/119) | 53.8% (64/119) | 10.7 ± 6.8 | 43 Ts, 76 Tap | 53.0 ± 11.6 |
| Alperi 2020 | 3 | 79.3 ± 3.2 | 33.3% (1/3) | 33.3% (1/3) | 100% (3/3) | 66.7% (2/3) | 0% (0/3) | 100% (3/3) |  | 3 Ts | 52.7 ± 2.5 |
| Altisent 2015 | 3 | 71.0 ± 8.7 | 66.6% (2/3) |  | 100% (3/3) | 66.7% (2/3) | 33.3% (1/3) | 100% (3/3) | 9.3 ± 2.5 | 3 Tap | 28 ± 1 |
| Bapat 2018 | 50 | 72.6 ± 9.4 | 58% (29/50) | 42% (21/50) | 68% (34/50) | 58% (29/50) | 16% (8/50) |  | 6.4 ± 5.5 | 50 Tap | 43.4 ± 11.8 |
| Bourguignon 2014^**^ | 430 |  |  |  |  |  |  |  |  | N/A |  |
| Brener 2024 | 126 | 75.2 ± 8.0 | 71.4% (90/126) | 45.2% (57/126) | 50.0% (63/126) | 46.8% (58/126) | 13.5% (17/126) | 47.5% (58/126) | 8.6 ± 5.9 | 126 Tat | 60.2 ± 9.7 |
| Brennan 2012 | 53 |  |  |  |  |  |  |  |  | N/A |  |
| Butnaru 2013 | 149 | 69.2 ± 9.9 | 53.6% (80/149) | 30.8% (46/149) |  | 37.6% (56/149) |  | 20.1% (30/149) |  | N/A |  |
| Capretti 2016 | 70 |  |  |  |  |  |  |  |  | 70 Ts |  |
| Cheung 2013 | 23 | 81.1 ± 5.8 | 39.1% (9/23) | 17.3% (4/23) |  | 60.9% (14/23) | 34.7% (8/23) | 56.5% (13/23) | 12.6 ± 6.9 | 23 Tap | 54.5 ± 12.3 |
| Conradi 2024 | 191 | 74.1 ± 8.0 | 62.8% (120/191) | 27.7 (53/191) | 68.1% (130/191) | 34.0% (65/191) | 14.1% (27/191) | 58.1% (111/191) | 7.7 ± 6.6 | 191 Tap | 44.7 ± 8.8 |
| Da Costa 2020 | 50 | 64.8 ± 9.8 | 28% (14/50) | 14% (7/50) | 18% (9/50) | 80% (40/50) | 18% (9/50) | 56% (28/50) | 8.3 ± 6.0 | 50 Tap | 59.2 ± 10.2 |
| Dahle 2017 | 11 | 64.3 ± 8.8 | 72.7% (8/11) | 18.1% (2/11) | 36.3% (4/11) | 55% (6/11) |  |  | 10.7 | 11 Tap | 37 ± 10 |
| Duncan 2017 | 5 | 73.8 ± 8.8 | 60% (3/5) |  | 40% (2/5) |  |  |  | 15.4 ± 5.5 | 5 Tap | 48 ± 9.1 |
| El Beze 2024 | 156 | 65.0 ± 18.5 | 34% (53/156) | 17.9% (28/156) | 25.6% (40/156) | 65.4% (102/156) |  | 56.1% (87/156) |  | 156 Ts | 56.7 ± 10.4 |
| Eleid 2017 | 87 | 75.1 ± 10.3 | 44.8% (39/87) |  |  | 69.9% (53/87) | 9.1% (8/87) |  | 12.8 | 80 Ts | 56.7 ± 13.2 |
| Eng 2017 | 13 | 75.0 ± 6.0 | 38.4% (5/13) | 38.4% (5/13) | 61.5% (8/13) | 70% (10/13) | 15.3% (2/13) |  | 10.9 ± 9.4 | 12 Ts | 49 ± 12 |
| Gaia 2017 | 12 | 61.7 ± 9.9 | 8.3% (1/12) | 41.7% (5/12) |  | 66.7% (8/12) |  |  | 15.8 ± 13.4 | 12 Tap | 66.7 ± 7.1 |
| Guerrero 2018 | 116 | 73.0 ± 12.0 | 25.5% (27/106) |  |  | 42.8% (42/98) |  | 53.2% (57/107) |  | 47 Ts, 46 Tap, 23 Tat | 60.0 ± 10.3 |
| Guerrero 2020^***^ | 780 | 76.1 ± 4.3 | 40.1% (313/780) | 27.8% (217/780) |  | 65% (597/903) |  | 49.9% (451/903) | 10.1 | 327 Ts, 360 Tap, 3 Tat, 90 unknown | 56.9 ± 3.3 |
| Guerrero 2023 | 91 | 74.5 ± 2.7 | 42.8% (39/91) | 29.6% (27/91) |  | 56.0% (51/91) | 13.1% (12/91) | 26.4% (24/91) | 7.7 | 75 Ts, 15 Tap | 54.6 ± 18.2 |
| Guerrero 2024 | 820 | 72.2 ± 10.4 | 49.1% (403/820) | 28.9% (237/819) |  | 70.6% (579/820) | 14.6% (120/820) | 57.9% (470/813) | 8.2 ± 6.9 | 770 Ts, 37 Tap, 2 Tat, 11 unknown | 47.8 ± 14.2 |
| Guimarães 2020 | 1005 | 59.3 ± 12.1 | 39.6% (398/1005) | 13.7% (138/1005) |  | 100% (1005/1005) | 15.3% (155/1005) | 1.8% (18/1005) |  | N/A |  |
| Gwak 2012 | 239 | 67.2 ± 11.2 | 25.1% (60/239) | 27.1% (65/239) |  | 81.2% (194/239) |  | 18.4% (44/239) |  | N/A | 58.6 ± 10.3 |
| Hosoba 2020 | 53 | 71.6 ± 8.9 | 49.1% (26/53) | 22.6% (12/53) |  |  |  | 0% (0/53) |  | N/A |  |
| Kalil 2021 | 31 |  |  |  |  |  |  |  |  |  |  |
| Kawano 2021 | 11 | 74.3 ± 9.0 | 18.1% (2/11) | 54.5% (6/11) | 18.1% (2/11) | 36% (4/11) | 27.2% (3/11) | 36.4% (4/11) | 9.1 ± 6.5 | 10 Tat | 64.4 ± 7.9 |
| Kuohn 2022 | 57 | 73.0 ± 12.0 | 35% (20/57) |  |  | 52% (29/57) |  |  |  |  |  |
| Long 2018 | 24 | 68.7 ± 14.2 | 50% (12/24) | 20.8% (5/24) |  | 75% (18/24) |  | 33.3% (8/24) | 8.9 ± 5.2 | 17 Ts, 7 Tap | 42.5 ± 14.1 |
| Ludwig 2021 | 11 | 77.0 ± 2.8 | 27.2% (3/11) | 9% (1/11) |  | 81.8% (9/11) |  | 90/9% (10/11) | 4.1 | 11 Tap | 45.0 ± 14.4 |
| Ludwig 2023 | 400 | 77.0 ± 2.6 | 59.5% (238/400) | 28.5% (114/400) | 55% (220/400) | 66.2% (233/400) | 12.2% (49/400) | 73.7% (286/400) | 5.9 | 33 Ts, 367 Tap | 45.0 ± 5.0 |
| Malaisrie 2024 | 50 | 70.1 ± 9.7 | 46.0% (23/50) | 18.0% (9/50) | 32.0% (6/50) | 52% (26/50) | 2.0% (1/50) |  | 4.1 ± 1.6 | 50 Ts |  |
| Mandiye 2022 | 37 |  |  |  |  | 61.2% (30/49) |  |  |  | N/A |  |
| Praz 2018 | 26 | 78.0 ± 7.0 | 7.6% (2/26) | 42.3% (11/26) | 30.7% (8/26) | 26.9% (7/26) |  | 69% (18/26) | 9.4 ± 4.8 | 26 Tat |  |
| Regueiro 2017 | 13 | 71.0 ± 8.0 | 76.9% (10/13) | 38.4% (5/13) | 76.9% (10/13) | 61.5% (8/13) |  | 92.3% (12/13) | 7.2 ± 3.6 | 13 Tap | 34 ± 9 |
| Rogers 2023 (1 and 2) | 197 | 76.6 ± 7.1 | 49.2% (100/203) | 42.8% (87/203) | 65% (132/203) |  | 5.4% (11/203) | 51.3% (101/197) | 6.8 | 197 Tap | 52.5 ± 10.6 |
| Schneider 2023 | 30 | 75.6 ± 8.0 | 73.3% (22/30) | 43.3% (13/30) |  | 70% (21/30) | 70% (21/30) | 3.3% (1/30) | 5.5 | 30 Ts | 45.3 ± 4.5 |
| Sorajja 2019 | 9 | 77.0 ± 6.0 | 55.5% (5/9) | 66.6% (6/9) | 66.6% (6/9) |  | 0% (0/9) | 78% (7/9) | 7.4 ± 3.6 | 9 Tap | 56 ± 8 |
| Ussia 2017 | 4 | 73.8 ± 2.9 | 100% (4/4) | 50% (2/4) |  | 50% (2/4) | 25% (1/4) | 100% (4/4) |  | 2 Ts, 2 Tap | 35 ± 6 |
| Webb 2019 | 10 | 76.1 ± 5.5 | 50% (5/10) |  |  | 30% (3/9) | 10% (1/10) | 40% (4/10) | 3.8 ± 2.5 | 9 Ts | 45.5 ± 13.5 |
| Webb 2020 | 14 | 84.0 ± 3.5 | 69.2% (9/13) | 15.3% (2/13) | 69.2% (9/13) | 92.9% (13/14) | 0% (0/13) | 64.3% (9/14) | 4.6 | 14 Ts | 51.8 ± 8.7 |
| Wilbring 2014 | 14 | 75.0 ± 5.0 | 46.1% (6/13) | 61.5% (8/13) | 61.5% (8/13) | 61.5% (8/13) | 23% (3/13) |  | 11.59 ± 3.10 | 13 Tap, 1 Tat | 44.5 ± 17.4 |
| Wild 2022 | 108 | 75.0 ± 7.0 | 57.4% (62/108) |  | 62.9% (68/108) | 70% (76/108) | 15.7% (17/108) | 78% (84/108) | 7.2 ± 5.3 | 104 Tap | 48 ± 12 |
| Ye 2015 | 31 | 78.7 ± 8.8 | 41.9% (13/31) | 22.5% (7/31) | 51.6% (16/31) |  | 32.2% (10/31) |  | 9.7 |  | 52.5 ± 13 |
| Yoon 2019 | 411 | 72.6 ± 11.9 | 45.8% (239/521) | 23.8% (124/521) |  |  | 15.7% (82/521) |  | 9.0 ± 7.0 | 206 Ts, 310 Tap, 5 Tat | 52.6 ± 13.7 |
| Zahr 2023 | 33 | 80.0 ± 2.5 | 63.6% (21/33) | 39.3% (13/33) | 27.2% (9/33) | 45.5% (15/33) |  |  | 5.3 | 33 Ts | 50.5 ± 5.7 |
| All studies | 6176 | 73.5 (95% CI 71.9-75.1) | 46.1% (2560/5555) | 26.4% (1366/5169) | 52.2% (852/1633) | 71.5% (3495/4886) | 15.4% (596/3870) | 42.0% (2009/4781) | 8.8 (7.5-9.7) | 1977 Ts, 1912 Tap, 196 Tat | 50.3 (47.4-53.1) |
| Comparison by valve intervention: SMVR vs TMVR | | | | | | | | | | | |
| All SMVR | 1966 | N/A | 39.0% (564/1446) | 18.0% (261/1446) | N/A | 89.9% (1285/1430) | 15.4% (155/1005) | 6.4% (92/1446) | N/A | N/A | N/A |
| All TMVR | 4210 | N/A | 48.6% (1996/4109) | 29.7% (1105/3723) | 52.2% (852/1633) | 63.9% (2210/3456) | 15.4% (441/2865) | 57.5% (1917/3335) | N/A | 1977 Ts, 1912 Tap, 196 Tat | N/A |
| Pooled SMVR estimate (95% CI)[^†^](https://www.nejm.org/doi/full/10.1056/NEJMoa2029603#fv-t1fn2) | 280 (23-539) | 66.8 (95%CI 61.5-72.0) | 0.47 (0.41-0.53) | 0.22 (0.16-0.31) | N/A | 0.94 (0.28-0.99) | N/A | 0.05 (0.01-0.23) | N/A | N/A | N/A |
| Pooled TMVR estimate (95% CI)[^†^](https://www.nejm.org/doi/full/10.1056/NEJMoa2029603#fv-t1fn2) | 108 (49-167) | 74.3 (95% CI 72.9-75.8) | 0.41 (0.30-0.52) | 0.31 (0.27-0.35) | 0.27 (0.19-0.37) | 0.60 (0.54-0.65) | 0.16 (0.11-0.21) | 0.58 (0.48-0.67) | 8.8 (7.5-9.7) | N/A | 50.1 (47.1-52.9) |
| P value^[†](https://www.nejm.org/doi/full/10.1056/NEJMoa2029603#fv-t1fn2)^ | 0.201 | 0.007 | 0.334 | 0.082 | N/A | 0.207 | N/A | 0.001 | N/A | N/A | N/A |
| Comparison by oral anticoagulation (OAC): OAC vs no OAC | | | | | | | | | | | |
| All OAC | 2760 | N/A | 39.8% (734/1846) | 21.0% (350/1670) | 46.6% (247/530) | 83.0% (1347/1623) | 14.2% (209/1469) | 18.4% (294/1594) |  | 332 Ts, 281 Tap, 36 Tat |  |
| All no OAC | 304 | N/A | 100% (4/4) | 50% (2/4) | N/A | 50% (2/4) | 25% (1/4) | 100% (4/4) |  | 2 Ts, 2 Tap |  |
| Pooled OAC estimate (95% CI)[^†^](https://www.nejm.org/doi/full/10.1056/NEJMoa2029603#fv-t1fn2) | 77 (21-132) | 73.1 (95% CI 69.9-76.3) | 0.43 (0.35-0.52) | 0.27 (0.19-0.37) | 0.48 (0.31-0.66) | 0.71 (0.46-0.87) | 0.11 (0.05-0.23) | 0.55 (0.29-0.79) | 6.8 (5.0-9.7) | N/A | 49.4 (43.6-55.1) |
| Pooled no OAC estimate (95% CI)[^†^](https://www.nejm.org/doi/full/10.1056/NEJMoa2029603#fv-t1fn2) | 18 (5-31) | N/A^‡^ | N/A^‡^ | N/A^‡^ | N/A^‡^ | N/A^‡^ | N/A^‡^ | N/A^‡^ | N/A^‡^ | N/A | N/A^‡^ |
| P value[^†^](https://www.nejm.org/doi/full/10.1056/NEJMoa2029603#fv-t1fn2) | 0.043 | N/A^‡^ | N/A^‡^ | N/A^‡^ | N/A^‡^ | N/A^‡^ | N/A^‡^ | N/A^‡^ | N/A^‡^ | N/A | N/A^‡^ |
| Comparison by anticoagulation regimen: DOACs vs VKAs | | | | | | | | | | | |
| All DOAC | 592 | N/A | 38.2% (203/532) | 14.7% (78/532) | 6.0 (32/532) | 97.6% (519/532) | 12.6% (63/500) | 3.7% (20/537) | N/A | 32 Ts | N/A |
| All VKA | 1759 | N/A | 43.7% (520/1191) | 24.3% (278/1146) | 25.9% (260/1003) | 76.1% (812/1067) | 12.4% (120/969) | 26.2% (278/1062) | N/A | 300 Ts, 281 Tap, 36 Tat | N/A |
| Pooled DOAC estimate (95% CI)[^†^](https://www.nejm.org/doi/full/10.1056/NEJMoa2029603#fv-t1fn2) | 59 (37-155) | 59.4 (95% CI 59.2-59.6) | 0.38 (0.34-0.42) | 0.15 (0.12-0.18) | 0.09 (0.04-0.20) | 0.99 (0.02-1.00) | N/A | 0.07 (0.01-0.42) | N/A | N/A | 53.7 (50.1-57.3) |
| Pooled VKA estimate (95% CI)[^†^](https://www.nejm.org/doi/full/10.1056/NEJMoa2029603#fv-t1fn2) | 53 (23-84) | 72.4 (95% CI 69.4-75.4) | 0.41 (0.38-51) | 0.29 (0.21-0.40) | 0.42 (0.23-0.65) | 0.69 (0.45-0.86) | 0.10 (0.04-0.22) | 0.56 (0.30-0.79) | 6.8 (5.0-9.7) | N/A | 49.5 (43.7-55.3) |
| P value[^†^](https://www.nejm.org/doi/full/10.1056/NEJMoa2029603#fv-t1fn2) | 0.909 | 0.001 | 0.529 | 0.001 | 0.003 | 0.332 | N/A | 0.027 | N/A | N/A | 0.226 |

^*^CKD was defined as eGFR < 60 mL/min/1.73 m²

^**^Patients with active or previous infective endocarditis were excluded

^***^Includes valve-in-valve and valve-in mitral-annular-calcification cohorts, valve thrombosis outcomes for valve-in-ring patients with longer follow-up are included in Guerrero et al. 2024

[^†^](https://www.nejm.org/doi/full/10.1056/NEJMoa2029603#fv-t1fn2)Background characteristics between SMVR/TMVR, OAC/No OAC and DOAC/VKA subgroups were compared with meta-analysis of continuous variables (N, age, STS PROM score, LV EF) or proportions (male, diabetes, CAD, AF, stroke or TIA, CKD) using random-effects models and maximum-likelihood method to estimate between-study variance.

^‡^Background characteristics of no OAC patients were only reported by a single study (Ussia et al., 2017) which precluded a pooled comparison.
