# Supplementary material for: Valve thrombosis and antithrombotic therapy after bioprosthetic mitral valve replacement: a systematic review and meta-analysis
Source: Eur Heart J Cardiovasc Pharmacother. 2025 Feb 4;11(3):251–63. doi: 10.1093/ehjcvp/pvaf005 (PMC12046575; doi:10.1093/ehjcvp/pvaf005)
Supplement: pvaf005_Supplemental_Files [file pvaf005_supplemental_files.zip › Supplementary Material 4_Metaregression analysis.docx]

**Supplementary Table 2: Meta-Regression Analysis of the incidence of bMVT**

|  |  | Univariate | | | | | | Multivariate | | | | |
| --- | --- | --- | --- | --- | --- | --- | --- | --- | --- | --- | --- | --- |
| Variable | | **Number of studies** | **β** | **SE** | **Lower bound** | **Upper bound** | **P value** | **β** | **SE** | **Lower bound** | **Upper bound** | **P value** |
| Follow-up time | | 47 | -0.0024 | 0.0004 | -0.0032 | -0.0016 | <0.0001 | -0.0028 | 0.0009 | -0.0045 | -0.0011 | 0.0012 |
| Age | | 38 | 0.0270 | 0.0281 | -0.0281 | 0.0821 | 0.3364 |  |  |  |  |  |
| Sex (male) | | 38 | -0.0062 | 0.0022 | -0.0105 | -0.0018 | 0.0058 | 0.0179 | 0.024 | -0.0019 | 0.0377 | 0.0764 |
| Diabetes | | 31 | -0.0126 | 0.0048 | -0.0220 | -0.0032 | 0.0086 | -0.0416 | 0.0229 | -0.0865 | 0.0033 | 0.0694 |
| Prior stroke or TIA | | 23 | -0.0195 | 0.0075 | -0.0342 | -0.0049 | 0.0091 |  |  |  |  |  |
| CKD | | 30 | 0.0261 | 0.0099 | 0.0066 | 0.0456 | 0.0086 | 0.0144 | 0.0084 | -0.0021 | 0.0309 | 0.0881 |
| STS PROM score | | 28 | 0.0013 | 0.0968 | -0.1885 | -0.1885 | 0.1910 |  |  |  |  |  |
| LV EF | | 26 | -0.0203 | 0.0394 | -0.0975 | 0.0568 | 0.6054 |  |  |  |  |  |

β values correspond to 1-U increments of continuous variables.

Abbreviations as in Table 2.
