# Supplementary material for: Valve thrombosis and antithrombotic therapy after bioprosthetic mitral valve replacement: a systematic review and meta-analysis
Source: Eur Heart J Cardiovasc Pharmacother. 2025 Feb 4;11(3):251–63. doi: 10.1093/ehjcvp/pvaf005 (PMC12046575; doi:10.1093/ehjcvp/pvaf005)
Supplement: pvaf005_Supplemental_Files [file pvaf005_supplemental_files.zip › Supplementary Material 6_VKA INR targets.docx]

**Supplementary Figure 3:** Duration of VKA therapy and target international normalised ratio (INR) studies reporting the use of VKAs.

| Study | Number of patients on VKAs | VKA regimen after valve intervention^*^ | INR target |
| --- | --- | --- | --- |
| Alperi, 2020 | 3 | ≥ 3 months | N/A |
| Altisent, 2015 | 3 | ≥ 3 months | N/A |
| Bapat 2018 | 48 | ≥ 3 months^**^ | 2.5-3.5 |
| Brennan 2012 | 21 | ≥ 3 months | 2.0-3.0 |
| Butnaru 2013 | 149 | ≥ 3 months | 2.0-3.0 |
| Capretti 2016 | 70 | ≥ 3 months^**^ | 3.0 |
| Cheung 2013 | 16 | Long term^***^ | N/A |
| Conradi 2024 | 62 | ≥ 3 months | 2.5-3.5 |
| Dahle 2017 | 11 | N/A | N/A |
| Duncan 2017 | 3 | N/A | N/A |
| El Beze 2024 | 124 | ≥ 3 months^**^ | 2.0-3.0 |
| Eleid 2017 | 85 | Long term | 2.0-3.0 |
| Eng 2017 | 9 | ≥ 3 months | 2.0-3.0 |
| Guererro 2018 | 54 | N/A | N/A |
| Guimarães 2020 | 505 | Long term*** | 2.0-3.0 |
| Hosoba 2020 | 50 | ≥ 3 months** | N/A |
| Kalil 2021 | 23 | N/A | N/A |
| Kawano 2021 | 10 | ≥ 3 months^**^ | 2.0-3.0 |
| Kuohn 2022 | 17 | N/A | N/A |
| Ludwig 2021 | 11 | Long term | 2.5-3.5 |
| Malaisrie 2024 | 50 | ≥ 6 months^**^ | 2.0-3.0 |
| Mandiye 2022 | 37 | ≥ 3 months^**^ | 2.0-3.0 |
| Praz 2018 | 26 | Long term^****^ |  |
| Regueiro 2017 | 8 | N/A | N/A |
| Rogers 2023 (1 and 2) | 197 | ≥ 3 months | 2.5-3.5 |
| Schneider 2023 | 20 | ≥ 3 months | N/A |
| Sorajja 2019 | 8 | ≥ 6 months | N/A |
| Webb 2020 | 11 | N/A | N/A |
| Wilbring 2014 | 6 | Long term | 2.0-3.0 |
| Wild 2022 | 76 | N/A | N/A |
| Zahr 2023 | 15 | ≥ 6 months** | 2.0-3.0 |

^*^ VKA regimen after mitral valve intervention in the absence of other indications for oral anticoagulation

^**^ in combination with long term single antiplatelet agent

^***^ all patients had atrial fibrillation

^****^ in combination with long term single antiplatelet agent
