# Supplementary figures and images for: Valve thrombosis and antithrombotic therapy after bioprosthetic mitral valve replacement: a systematic review and meta-analysis
Source: Eur Heart J Cardiovasc Pharmacother. 2025 Feb 4;11(3):251–63. doi: 10.1093/ehjcvp/pvaf005 (PMC12046575; doi:10.1093/ehjcvp/pvaf005)

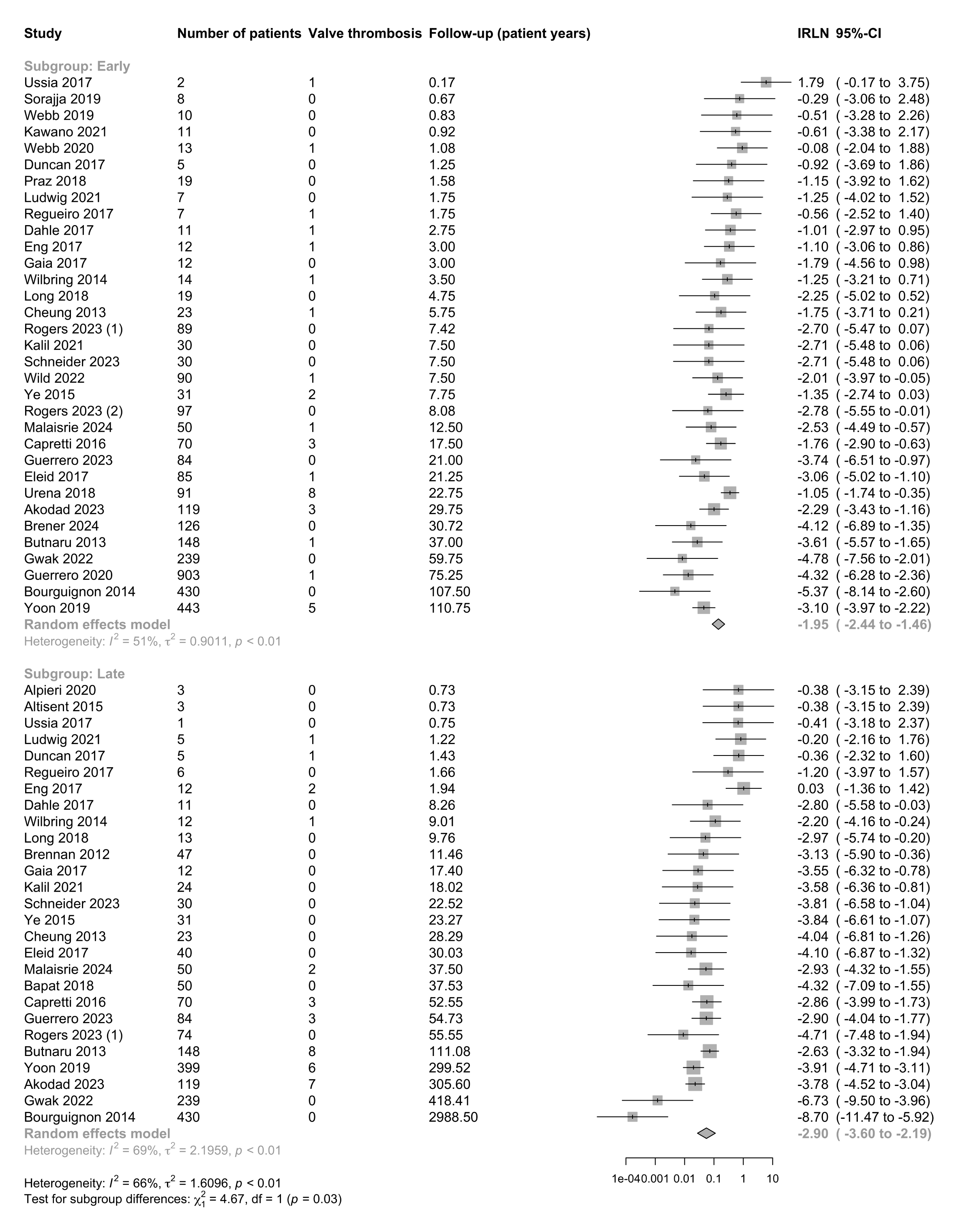

Supplement: pvaf005_Supplemental_Files [file pvaf005_supplemental_files.zip › Supplementary Material 5_Timing of bMVT_forest plot.tif]
